# Supplementary figures and images for: Structural basis for sarbecovirus Rc-o319 spike adaptation to Rhinolophus cornutus Bat ACE2 and constraints on switching to human ACE2
Source: PLoS Pathog. 2026 May 21;22(5):e1014245. doi: 10.1371/journal.ppat.1014245 (PMC13232947; doi:10.1371/journal.ppat.1014245)

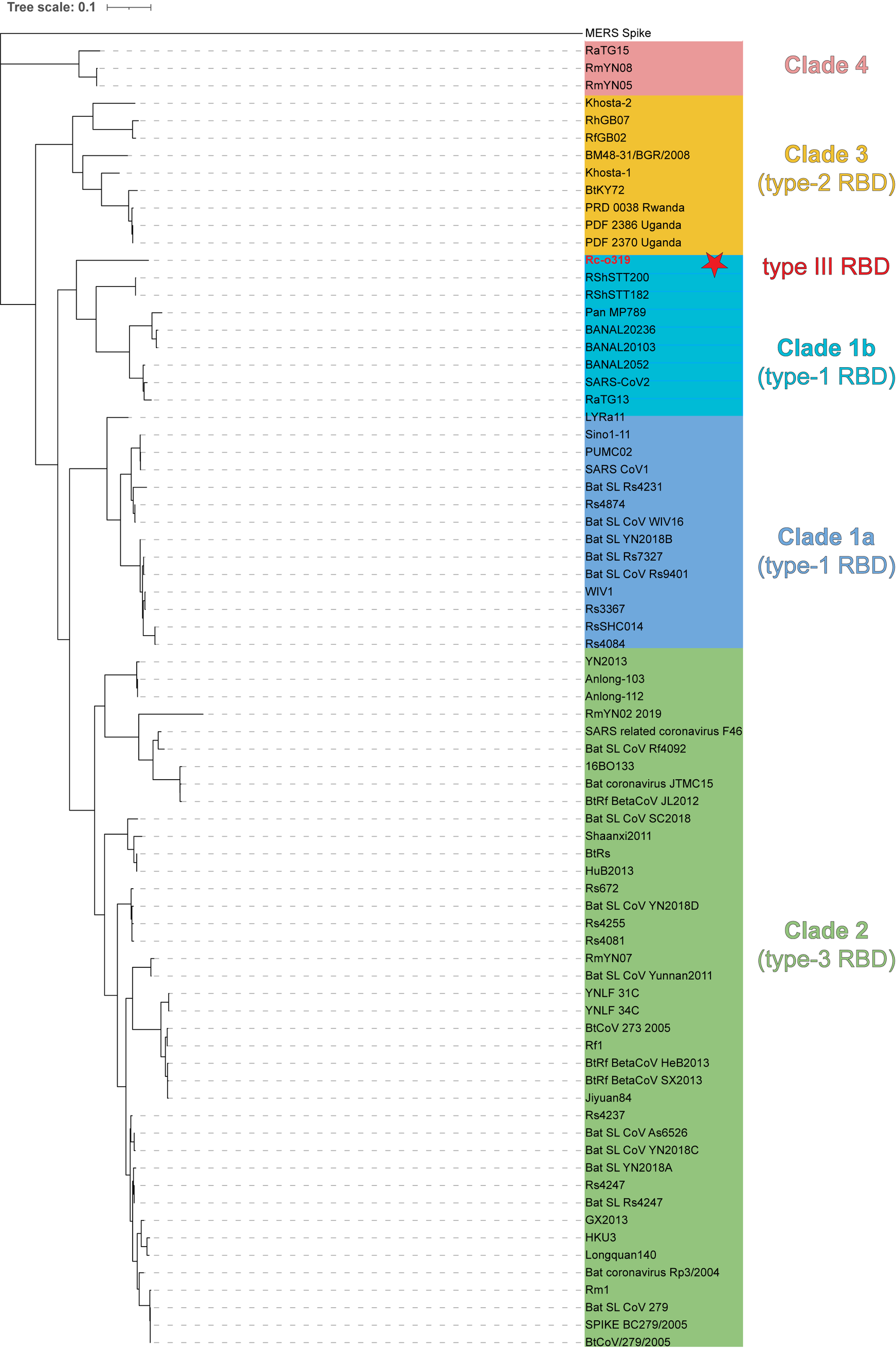

Supplement: S1 Fig — The tree shows the evolutionary relationships based on sequence similarities. (TIF) [file ppat.1014245.s001.tif]

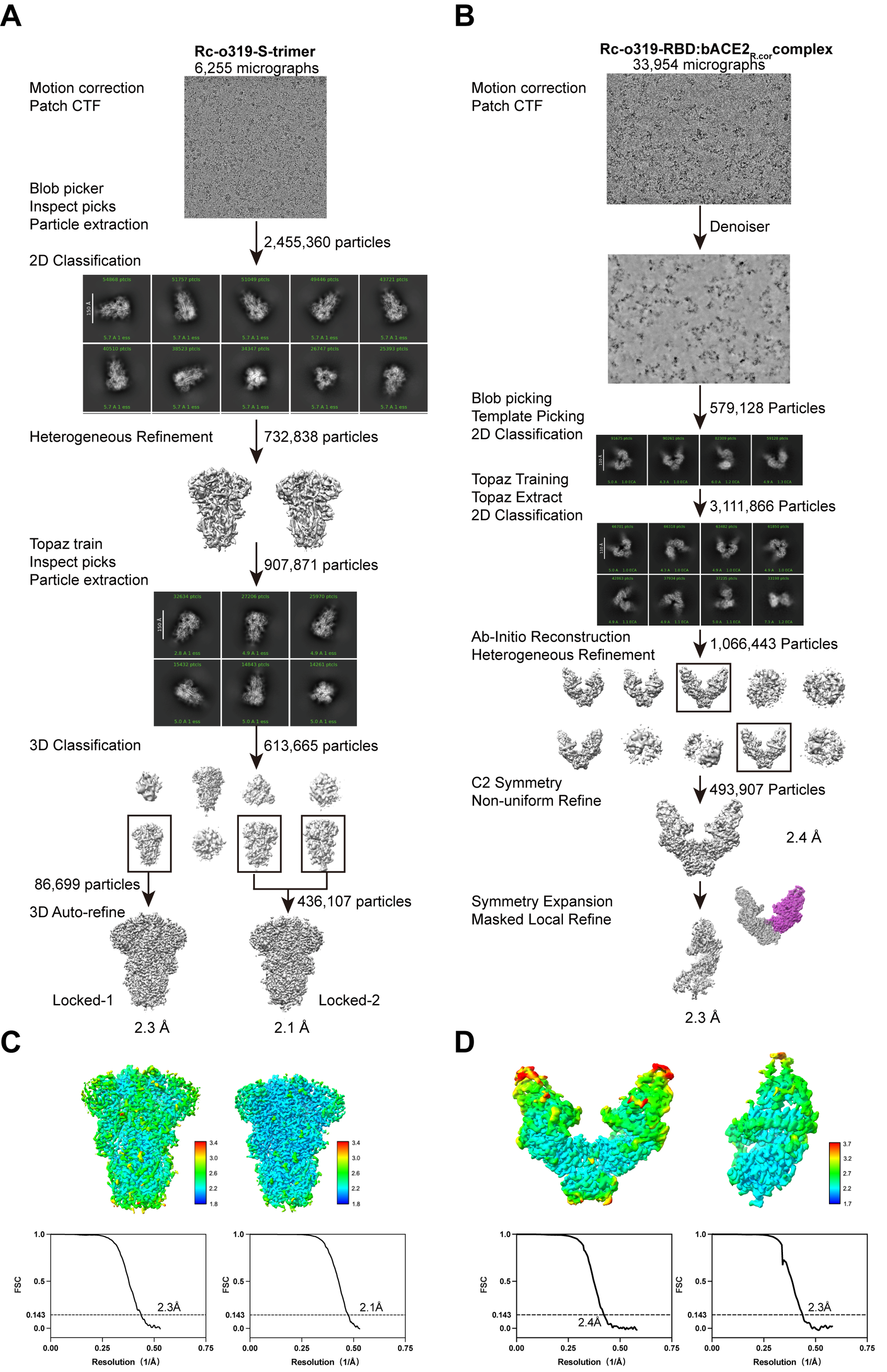

Supplement: S2 Fig — (A-B) Data processing pipelines are shown for the Rc-o319 S-trimer locked-1 and locked-2 structures and Rc-o319-RBD:bACE2R.cor structures. 3D and 2D classification steps were used to remove contaminating particles. Two conformations were identified in the 3D classification for the Rc-o319 S-trimer. (C-D) Local resolution maps for the Rc-o319 S-trimer and Rc-o319-RBD:bACE2R.cor complex structures (top panels) and global resolution assessments by Fourier shell correlation at the 0.143 criterion (bottom panels). (TIF) [file ppat.1014245.s002.tif]

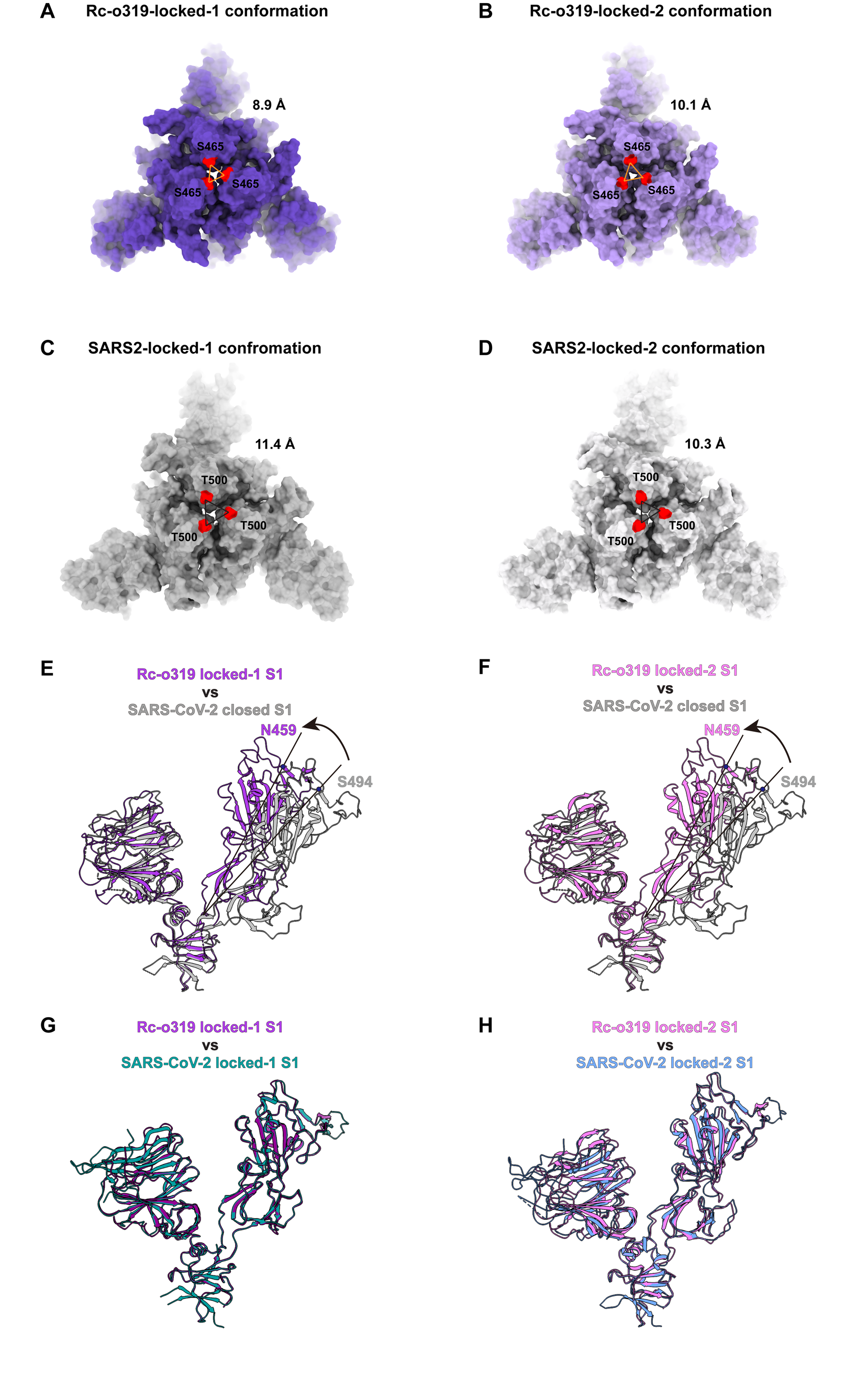

Supplement: S3 Fig — (A-B) Top-views of the two locked Rc-o319 S-trimer structures shown in molecular surface representation. Apex residues (S465Rc-o319) in each S-trimer are colored red. In each S-trimer structure, the distance between apices is indicated by triangles, with the distances indicated. (C-D) Top-views of locked-1 (PDB: 7XTZ) and locked-2 (PDB: 7XU2) SARS-CoV-2 S-trimer structures, apex positions (T500SARS2) are colored red, with the inter-apex distances indicated. (E-F) Comparison of the determined locked-1 and locked-2 Rc-o319 S1 structures (extracted from the Rc-o319 S-trimer structures) with the SARS-CoV-2 S1 structure in the closed conformation (gray, PDB: 7XU3). In the locked Rc-o319 S-trimers, the receptor-binding domains (RBDs) are positioned closer to the N-terminal domains (NTDs). (G-H) The S1 structures within the locked Rc-o319 S-trimers closely resemble those observed in the corresponding locked SARS-CoV-2 S-trimers (locked-1 PDB: 7XTZ, locked-2 PDB: 7XU2). (TIF) [file ppat.1014245.s003.tif]

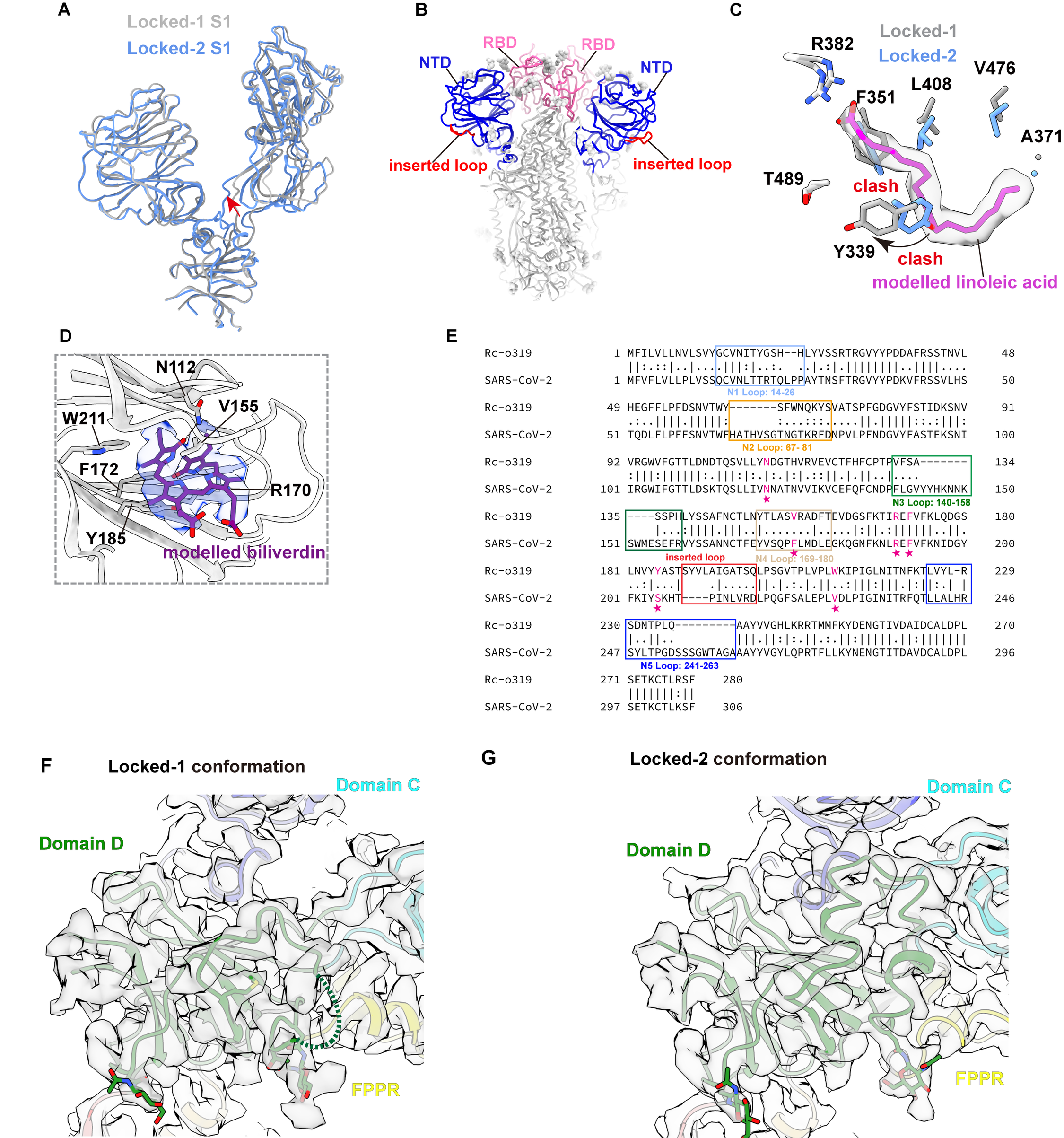

Supplement: S4 Fig — (A) Comparison of the locked-1 and locked-2 S1 structures of Rc-o319 S-trimer. (B) Rc-o319 locked-1 S-trimer structure showing the NTD and RBD. The “inserted loop” exposed to the exterior of the S-trimer is highlighted in red. (C) Comparison of the fatty-acid binding pockets of the fatty-acid-bound locked-1 and the fatty-acid-unbound locked-2 Rc-o319 S-trimer structures. A notable reorientation of the Y339Rc-o319 side-chain (highlighted by red labels) is observed between the occupied and unoccupied fatty-acid binding pockets. Due to the contraction of the fatty-acid binding pocket, the side-chains of Y339Rc-o319 and F351Rc-o319 in the unoccupied fatty-acid pocket would clash with the modelled linoleic acid molecule, rendering the unoccupied pocket incompatible with lipid binding. (D) Modelled biliverdin is shown in purple stick representation with its density (blue). Residues involved in hydrophobic, hydrogen-bonding, and cation-π interactions with the bound biliverdin are shown as sticks. (E) An alignment of Rc-o319 and SARS-CoV-2 N-terminal domain (NTD) amino acid sequences. Modelled biliverdin interacting residues are marked by purple stars in the alignment. N1–N5 loops are framed by boxes colored in light blue, orange, green, brown and blue, respectively. Additionally, a four-amino-acid insertion, forming the “inserted loop” in the Rc-o319 NTD by comparison with SARS-CoV-2 NTD, is framed by a red box. (F-G) Cryo-EM densities of Domain D and the surrounding regions in locked-1 and locked-2 conformations. In the locked-1 conformation, Domain D contains a large disordered Domain D-loop; In locked-2 conformation, Domain D is fully ordered, with the disordered Domain D-loop in locked-1 refolded into two short α-helices. (TIF) [file ppat.1014245.s004.tif]

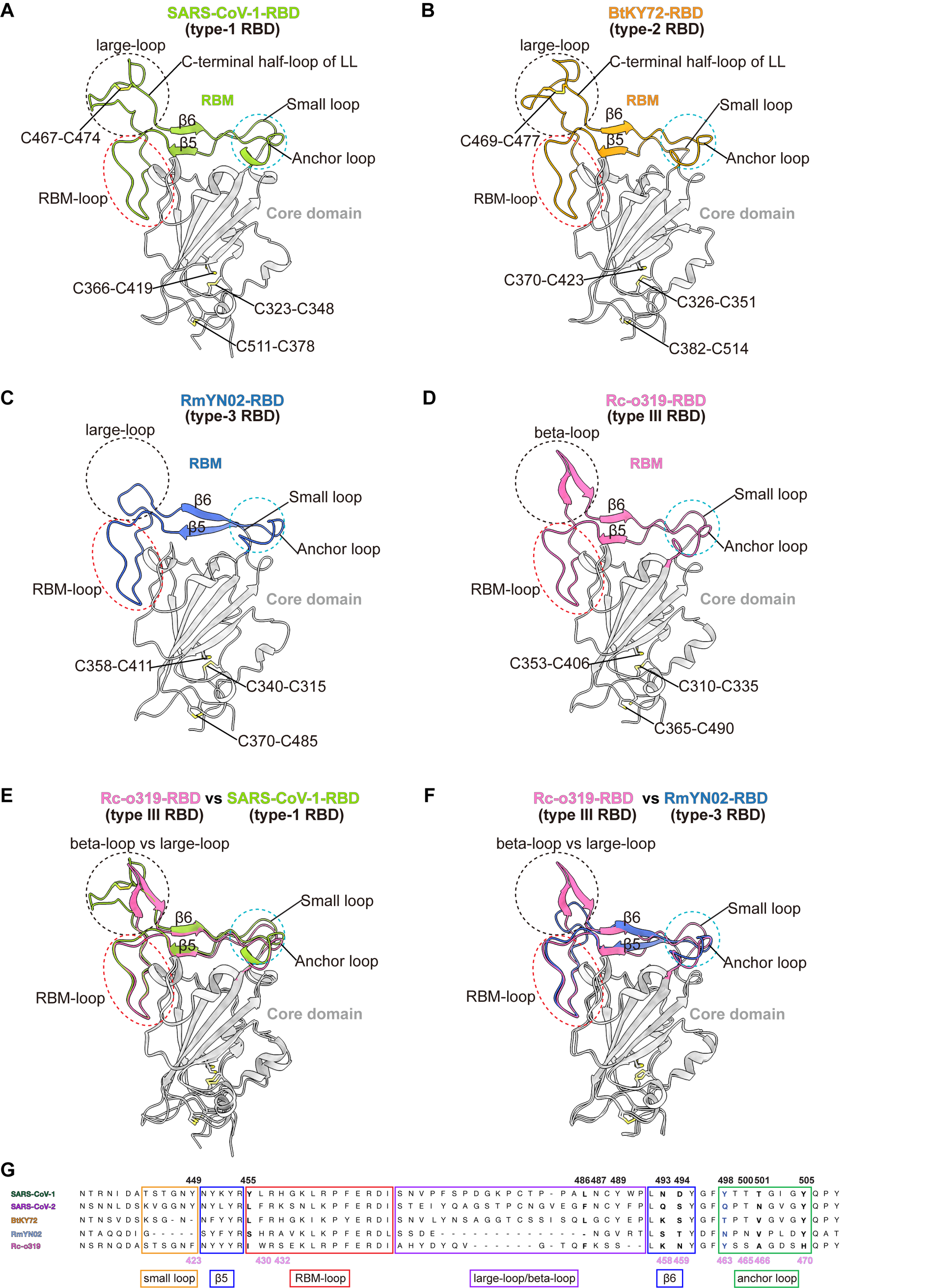

Supplement: S5 Fig — (A-D) Structures of representative type-1, type-2, type-3, and type III RBDs are shown in the same orientation. The RBM (receptor binding motif) regions are highlighted in colors, while the conserved RBD core domains are colored in gray. Black dashed circles are shown to mark the locations of the SL (Small-loop), AL (anchor-loop), and LL (large-loop) or beta-loop to highlight structural differences in the RBMs of different types of RBDs. Red dashed ovals highlight the RBM-loop structures. (E) Superimposition of the type III Rc-o319-RBD and the type-1 SARS-CoV-1-RBD structures. (F) Superimposition of the type III Rc-o319-RBD and the type-3 RmYN02-RBD structures. (G) An alignment of representative types 1–3 and type-III RBM amino acid sequences. Amino acid residue numbers are shown according to SARS-CoV-2 (black, top) and Rc-o319 (pink, bottom) sequences. (TIF) [file ppat.1014245.s005.tif]

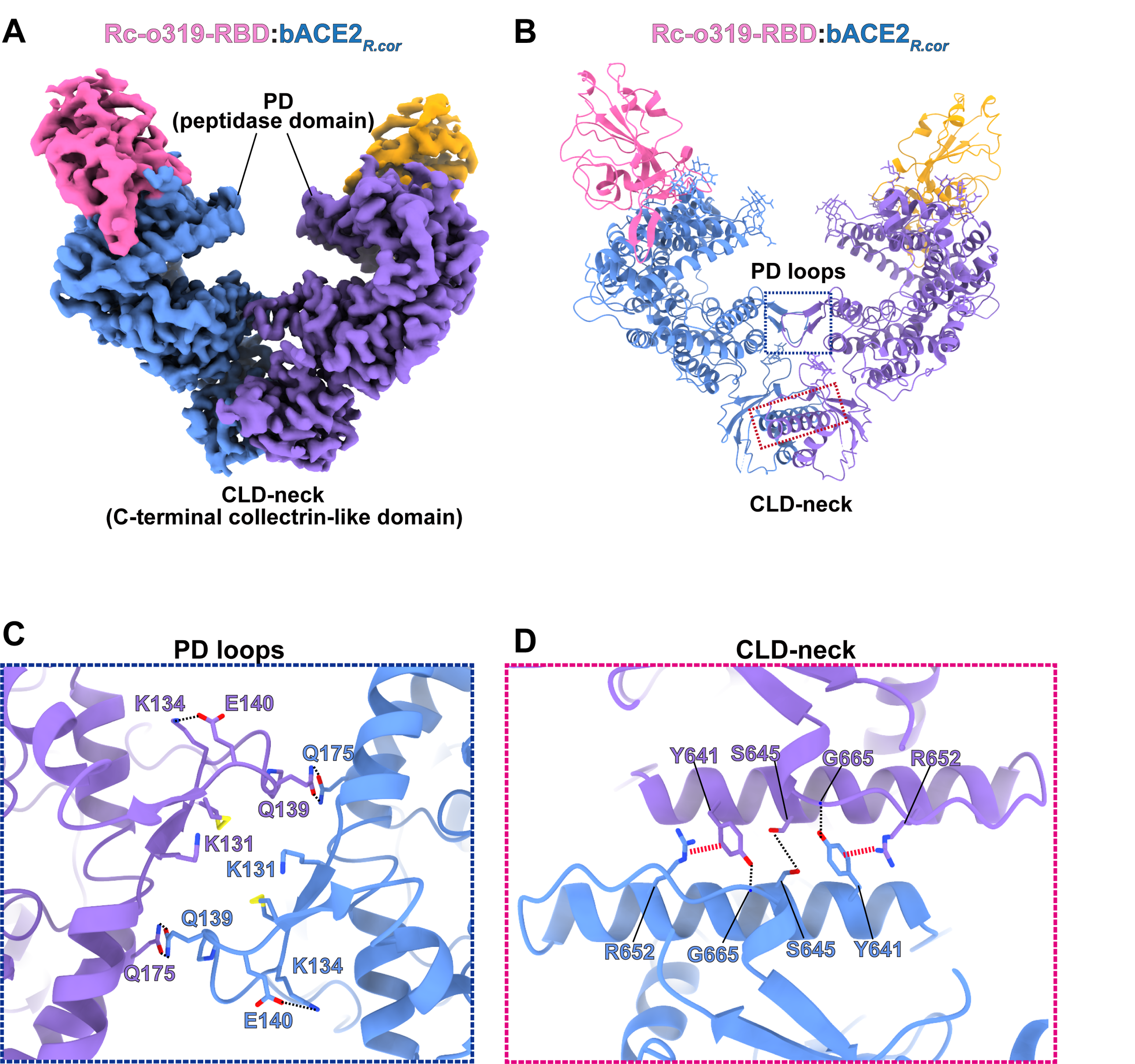

Supplement: S6 Fig — (A-B) Cryo-EM density (A) and the associated molecular model (B) of the Rc-o319-RBD:bACE2R.cor dimer. (C) Detailed dimer interface interactions in the peptidase domain (PD) loop region. (D) Detailed dimer interface interactions in the C-terminal collectrin-like domain (CLD) neck region. Hydrogen bonds are shown as black dashed lines, salt bridges are shown as blue dashed lines, and cation-π interactions are shown as red dashed lines. Dashed boxes in (B) indicate the locations of the interfaces shown in panels C and D. (TIF) [file ppat.1014245.s006.tif]

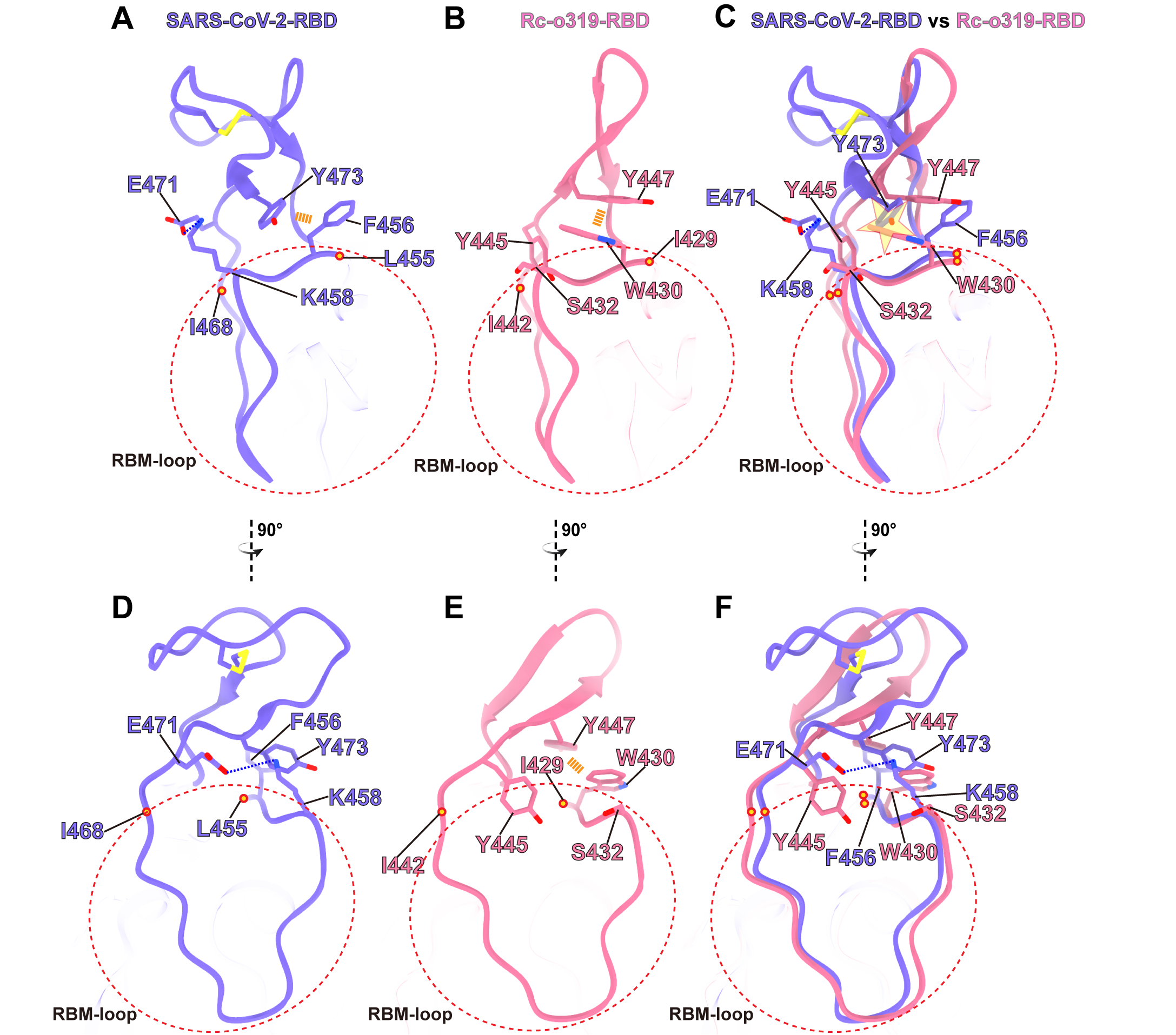

Supplement: S7 Fig — (A-B and D-E) The beta-loop-RBM-loop region of Rc-o319-RBD and the large-loop-RBM-loop region of SARS-CoV-2-RBD are shown in two different viewing angles. (A) Y473SARS2 of the SARS-CoV-2 large-loop and F456SARS2 of the RBM-loop form a pi-pi interaction. (B) The corresponding residues Y447Rc-o319 of the Rc-o319 beta-loop and W430Rc-o319 of the Rc-o319 RBM-loop also form a pi-pi interaction in a substantially different conformation. (C) A superposition of the Rc-o319 beta-loop-RBM-loop and the SARS-CoV-2 large-loop-RBM-loop structures. The superposition reveals a clash (highlighted by a yellow star) between the side-chains of Y473SARS2 and W430Rc-o319. (D) In the rotated view, E471SARS2 forms a salt-bridge with the RBM-loop residue K458SARS2, likely stabilizing the SARS-CoV-2 large-loop. (E) The corresponding residues in Rc-o319, Y445Rc-o319 of beta-loop and S432Rc-o319 of RBM-loop are not interacting. (F) The superposition of the Rc-o319 beta-loop-RBM-loop and the SARS-CoV-2 large-loop-RBM-loop structures in a rotated view. (TIF) [file ppat.1014245.s007.tif]

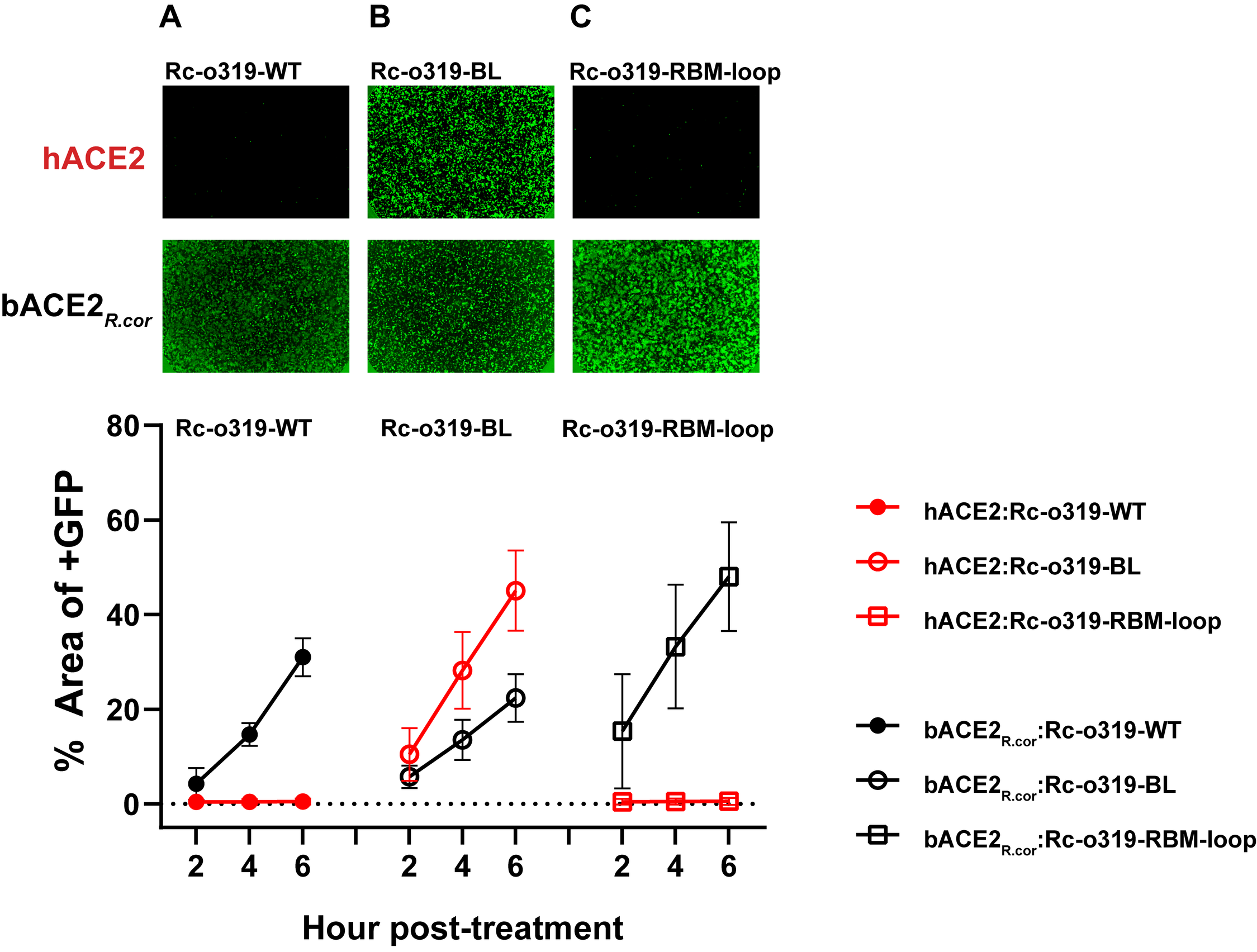

Supplement: S8 Fig — (A-C) Representative cell-cell fusion images captured at 6 hours post-cell-mixing are shown. Effector cells expressing WT and variant Rc-o319 S-proteins were tested against receptor cells expressing either bACE2R.cor or hACE2. The Bottom panel: Cell-cell fusion was quantified by assessing GFP+ areas at 2, 4, and 6 hours post-cell-mixing. (TIF) (PNG) [file ppat.1014245.s008.tif]

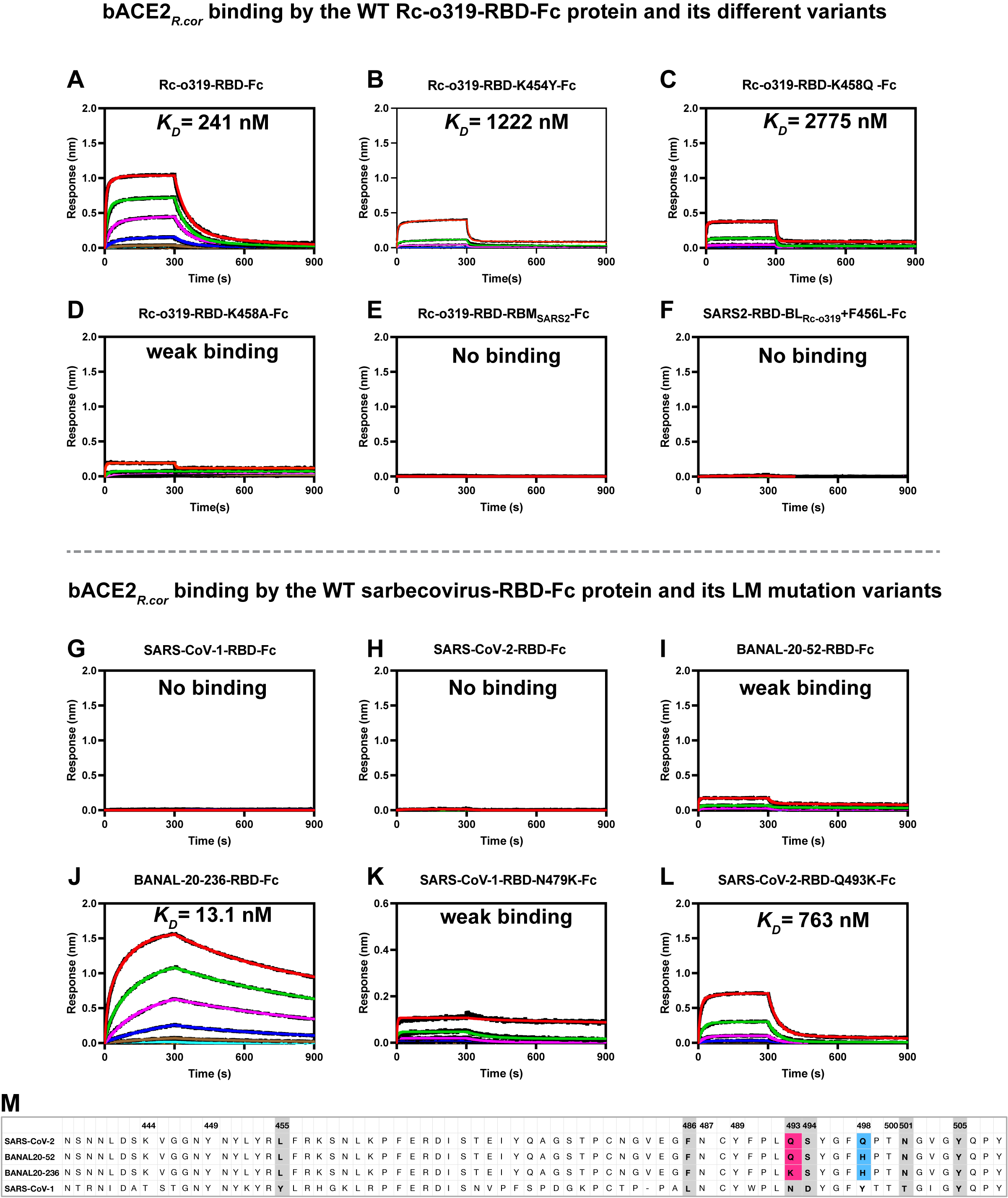

Supplement: S9 Fig — (A-E) bACE2R.cor binding by the WT Rc-o319-RBD-Fc protein and its different variants, including K454YRc-o319 in BL region (B) K458Q/ARc-o319 in LM (C and D), Rc-o319 RBD RBM exchanged for SARS-CoV-2 RBM (RBMSARS2) (E) and (F) SARS-CoV-2-RBD-Fc with LL exchanged for BL of Rc-o319 (BLRc-o319) with an extra F456LRc-o319 mutation in the RBM-loop. (G-J) bACE2R.cor binding by wild-type RBD-Fc proteins of SARS-CoV-1, SARS-CoV-2, BANAL-20-52, and BANAL-20-236. (K-L) bACE2R.cor binding by LM variants of SARS-CoV-1-RBD-Fc (N479KSARS1) and SARS-CoV-2-RBD-Fc (Q493KSARS2) proteins. (M) An alignment of SARS-CoV-2, BANAL-20-236, BANAL-20-52, and SARS-CoV-1 RBM amino acid sequences. Compared to the SARS-CoV-2 RBM, there are two amino-acid changes, Q493KSARS2 and Q498HSARS2, in BANAL-20-236 and one amino-acid change, Q498HSARS2, in BANAL-20-52. These changes likely favor bACE2R.cor binding by comparison with the SARS-CoV-2 Q493SARS2 and Q498SARS2 residues (H-J). (PNG) [file ppat.1014245.s009.png]

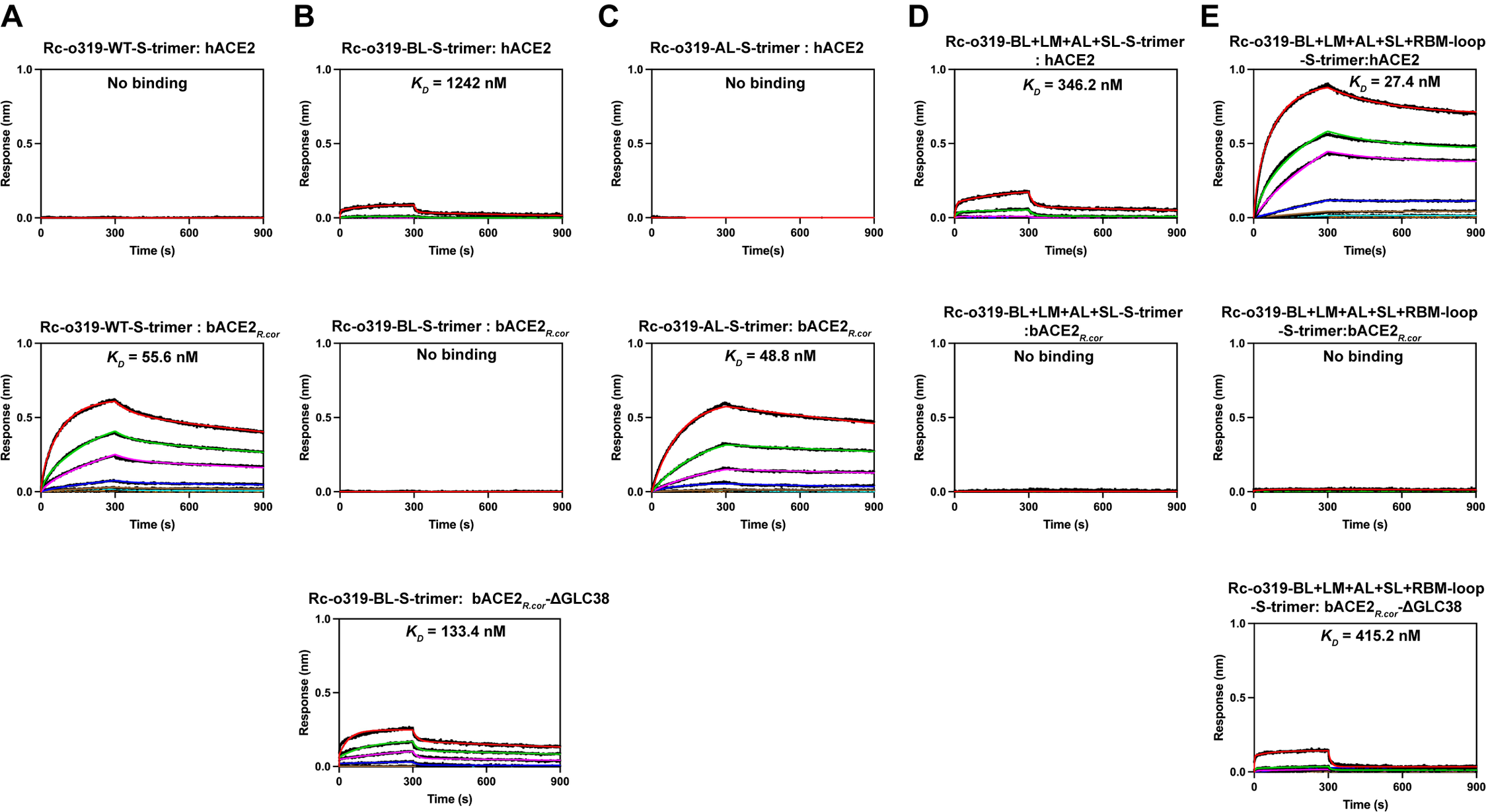

Supplement: S10 Fig — (A-E) Binding sensorgrams were recorded by immersing biosensors immobilized with hACE2 (first panels), WT bACE2R.cor (second panels), or bACE2R.cor-ΔGLC38 (third panels) into three-fold serial dilutions of S-trimer solutions, with concentrations ranging from 3000 to 4.09 nM. For WT (A) and the Rc-o319 AL S protein (C), concentrations ranged from 1500 to 2.04 nM. (TIF) [file ppat.1014245.s010.tif]

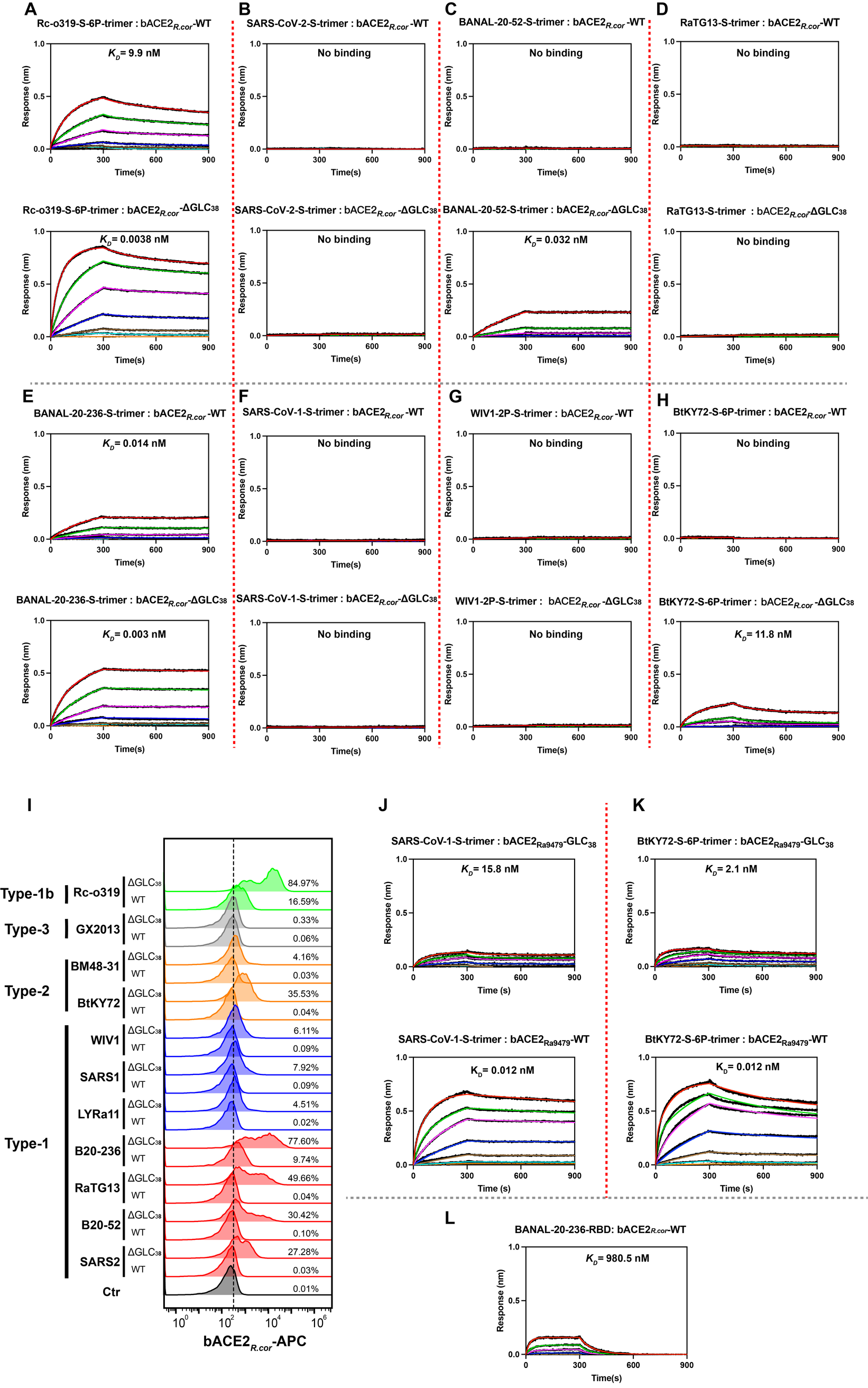

Supplement: S11 Fig — (A-H) Binding sensorgrams were recorded by submerging WT bACE2R.cor (top panels) or bACE2R.cor-ΔGLC38 (removing the glycan of Asn38 by Thr40Ala mutation, bottom panels) immobilized biosensors into 3-fold serially diluted S-trimer solutions, with concentrations ranging from 800 to 1.09 nM. (I) Binding of bACE2R.cor-WT or bACE2R.cor-ΔGLC38 by cell-surface expressed S-proteins as assessed by flow cytometry. bACE2R.cor-WT-Fc or bACE2R.cor-ΔGLC38-Fc protein was incubated with cells expressing S-proteins before ACE2 binding was quantified using a goat anti-human IgG-APC as the probe. (J-K) Binding of bACE2Ra9479-WT and bACE2Ra9479-GLC38 (introducing the Asn38-glycan by the Thr40Ala mutation) by S-trimers of SARS-CoV-1 and BtKY72 as assessed by BLI assays. (L) Binding of bACE2R.cor-WT by monomeric BANAL-20-236 RBD in BLI assays. Monomeric BANAL-20-236 RBD was threefold serially diluted from 3000 to 4.09 nM. Estimated KD values are shown next to their corresponding binding curves. (TIF) [file ppat.1014245.s011.tif]

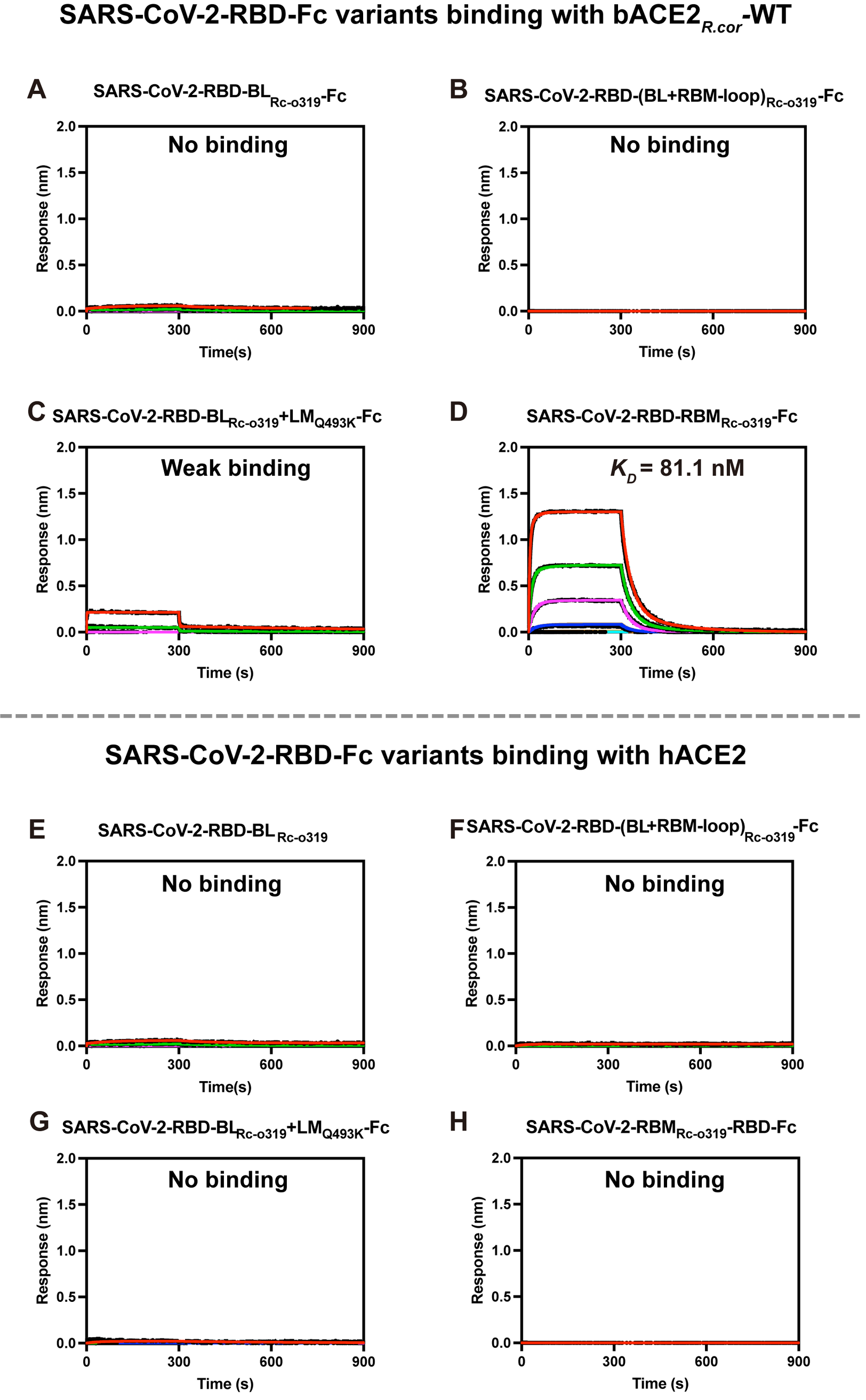

Supplement: S12 Fig — (A-D) bACE2R.cor binding by different variants of SARS-CoV-2-RBD-Fc, including LL swapped for the Rc-o319 BL region (SARS-CoV-2-RBD-BLRc-o319-Fc) (A), swapped for the Rc-o319 BL region and RBM-loop (B), LL swapped for the Rc-o319 BL region plus lamella Q493SARS2K mutation (SARS-CoV-2-RBD-BLRc-o319 + LMQ493K-Fc) (C), and the whole SARS-CoV-2 RBM swapped for the Rc-o319 RBM (SARS-CoV-2-RBD-RBMRc-o319-Fc) (D). (E-H) hACE2 binding by SARS-CoV-2-RBD-Fc variants, corresponding to those in A-D. Dimeric SARS-CoV-2-RBD-Fc proteins were immobilized on the BLI sensors and tested binding against dimeric Rhinolophus cornutus bat ACE2 (bACE2R.cor) or hACE2 protein as the analyte in solution. bACE2R.cor and hACE2 were threefold serially diluted from 3000 to 4.09 nM. Estimated KD values are shown next to their corresponding binding curves. (TIF) [file ppat.1014245.s012.tif]

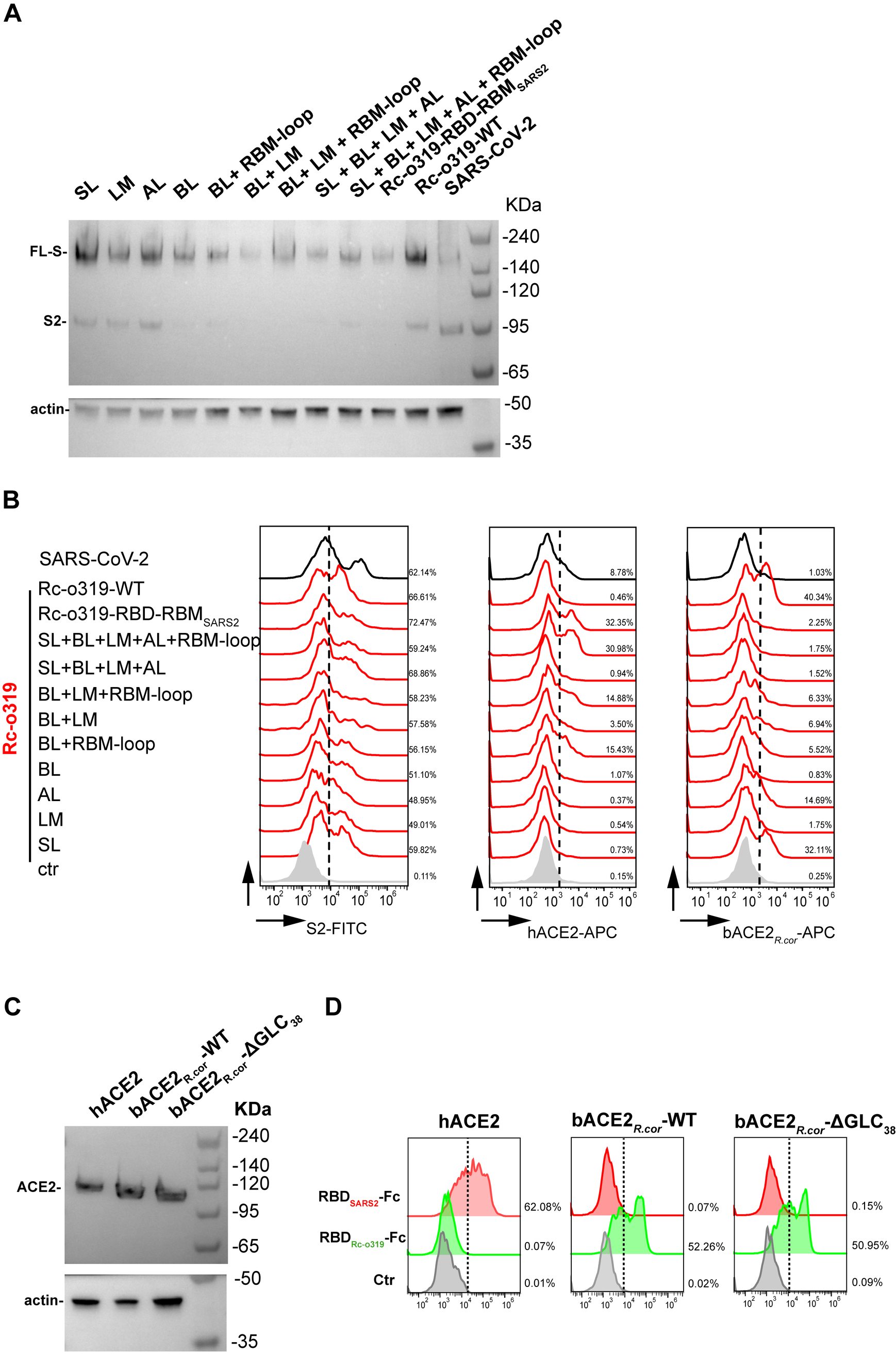

Supplement: S13 Fig — (A) Total cell S-protein expression was quantified by western blot. Lysates of cells expressing different S-proteins were probed using an anti-FLAG antibody with β-actin as the control. (B) Quantification of SARS-CoV-2 and Rc-o319 variant S-protein expression (left panels) and their ACE2 binding (middle panels, hACE2 binding; right panels, bACE2R.cor binding) using flow cytometry. (C) Total cell hACE2, bACE2R.cor and bACE2R.cor-ΔGLC38 protein expression was quantified using an anti-flag-tag antibody as the probe by western blot. (D) RBD-Fc binding by ACE2 expressing cells as detected by flow cytometry. Cells surface-expressing hACE2, bACE2R.cor-WT, or bACE2R.cor-ΔGLC38 were incubated with SARS-CoV-2 or Rc-o319 RBD-Fc proteins. Binding of dimeric RBD-Fc proteins was quantified using a goat anti-human IgG-APC as the probe. (TIF) [file ppat.1014245.s013.tif]

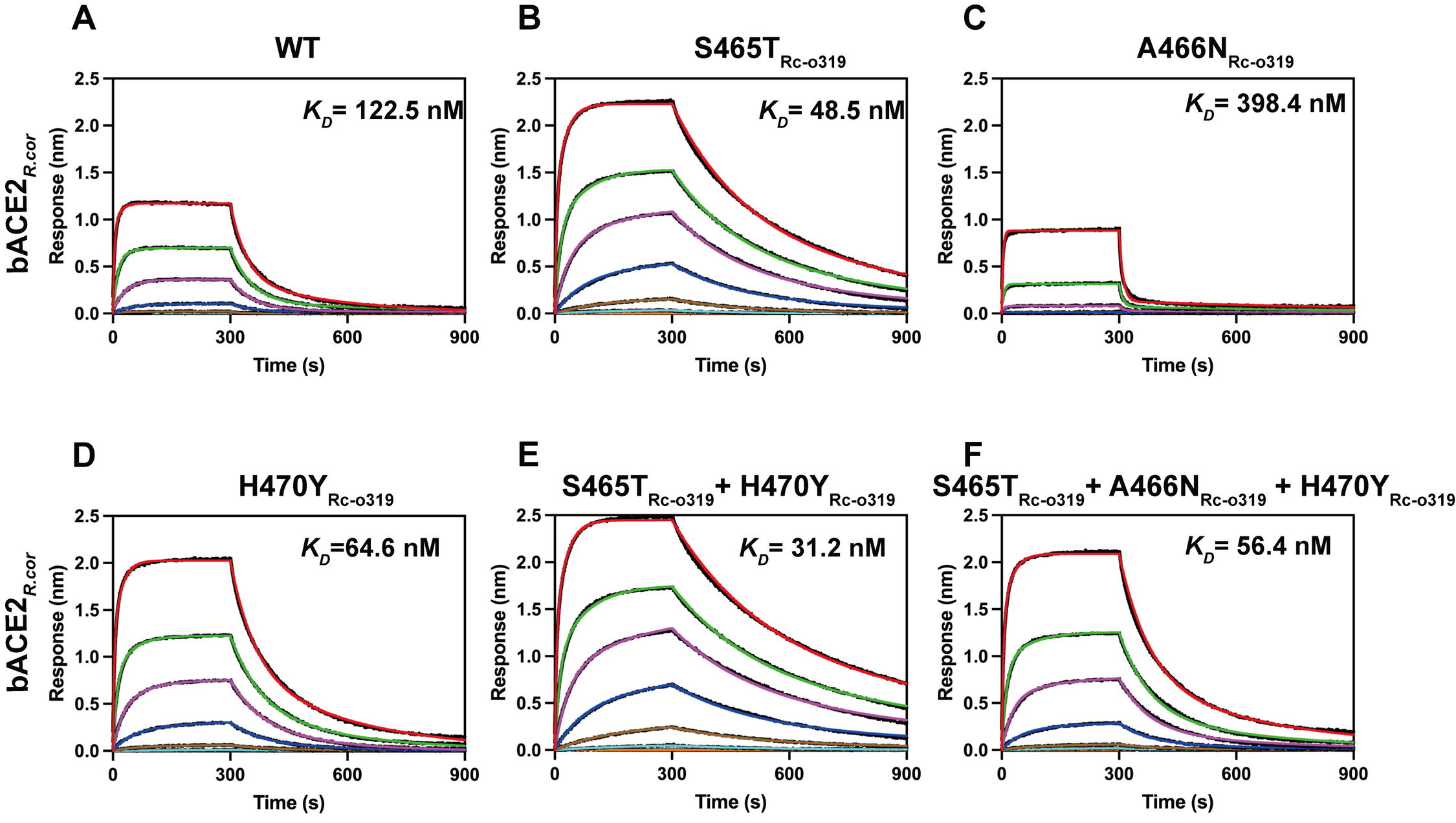

Supplement: S14 Fig — (A-F) Dimeric Rc-o319 RBD-Fc proteins were immobilized on BLI sensors, and dimeric bACE2R.cor was used as the analyte in solution, tested using three-fold serial dilutions ranging from 3000 to 4.09 nM. KD values estimated from each binding experiment are shown alongside the corresponding binding curves. (TIF) [file ppat.1014245.s014.tif]

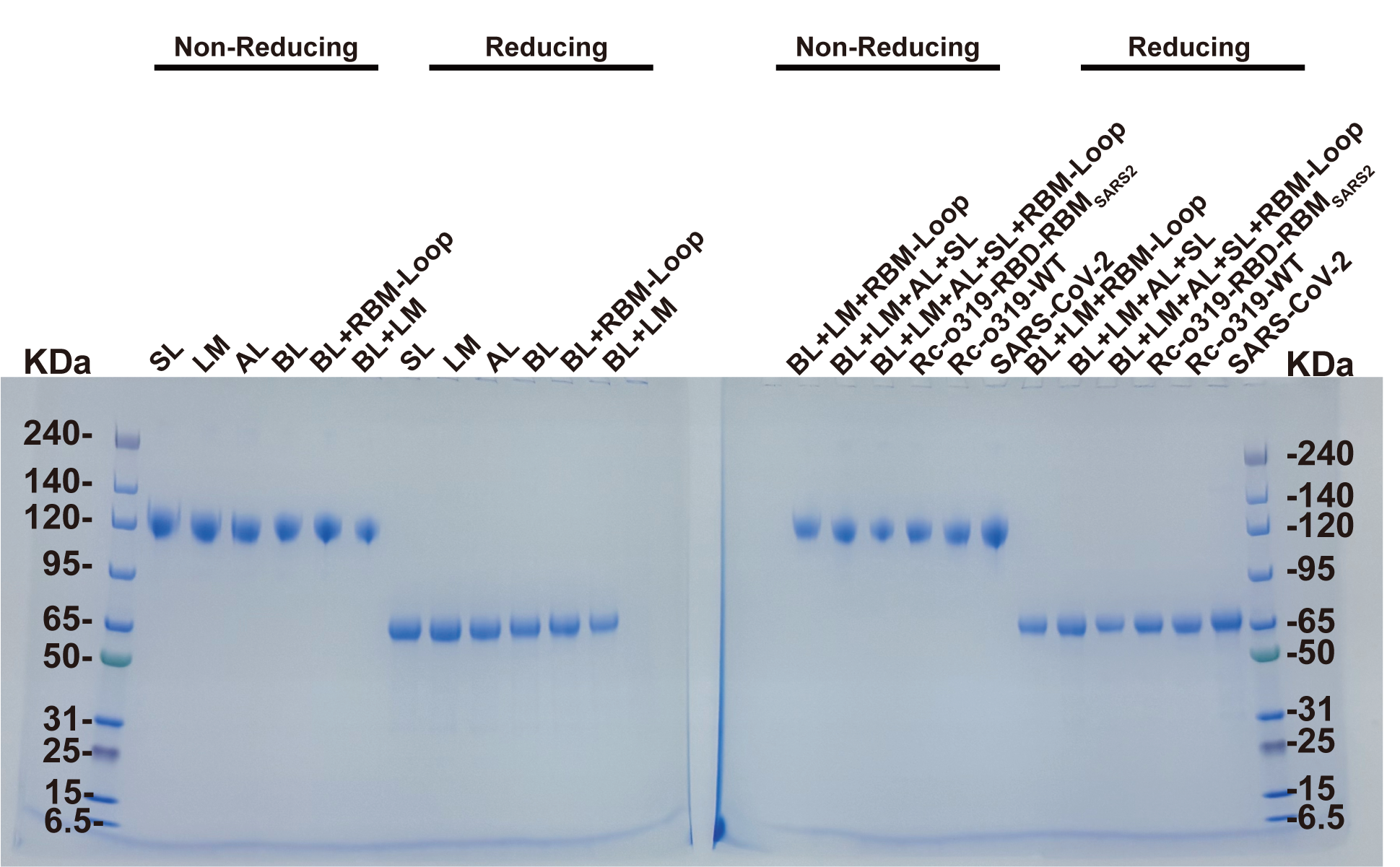

Supplement: S15 Fig — (TIF) [file ppat.1014245.s015.tif]

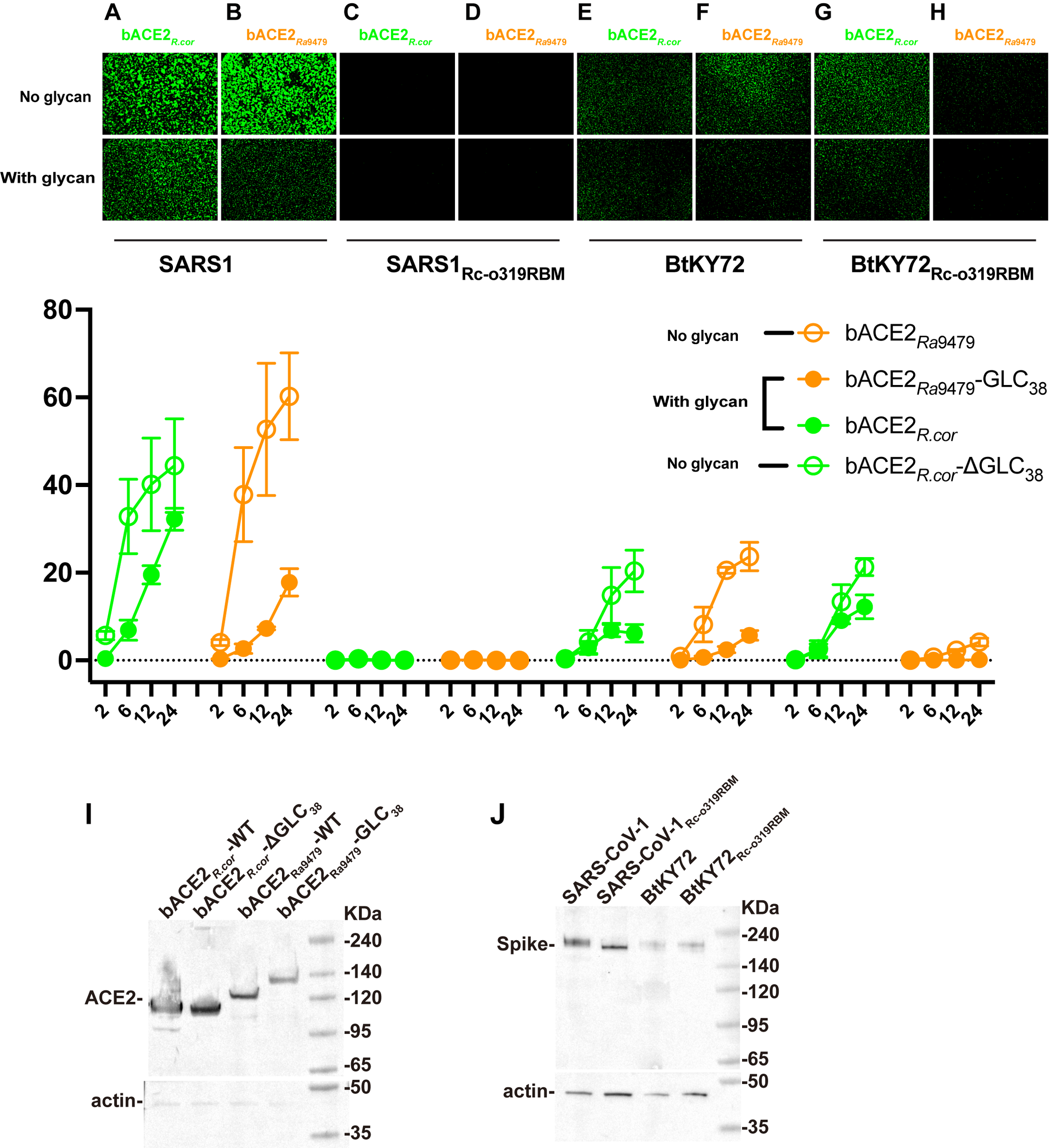

Supplement: S16 Fig — (A-H) The RBMs of BtKY72 and SARS-CoV-1 S-protein were replaced with that of Rc-o319, generating the chimeric SARS1Rc-o319RBM and BtKY72Rc-o319RBM S-proteins, respectively. In parallel, we also introduced an Asn38-glycan into bACE2Ra9479 by mutation Asp38 to Asn, yielding the bACE2Ra9479-GLC38 construct. Representative cell-cell fusion images captured at 12 hours post-cell-mixing are shown. Effector cells expressing S-proteins were tested against receptor cells expressing either wild-type or variant of bACE2R.cor or bACE2Ra9479. The bottom two panels: Cell-cell fusion was quantified by assessing GFP+ areas at 2, 6, 12 and 24 hours post-cell-mixing. (I) Total cell bACE2R.cor, bACE2R.cor-ΔGLC38, bACE2Ra9479 and bACE2Ra9479-GLC38 protein expression was quantified using an anti-flag-tag antibody as the probe by western blot. (J) Total cell S-protein expression of SARS-CoV-1, SARS1Rc-o319RBM, BtKY72 and BtKY72Rc-o319RBM was quantified using an anti-S2-tag antibody as the probe by western blot. (TIF) [file ppat.1014245.s016.tif]

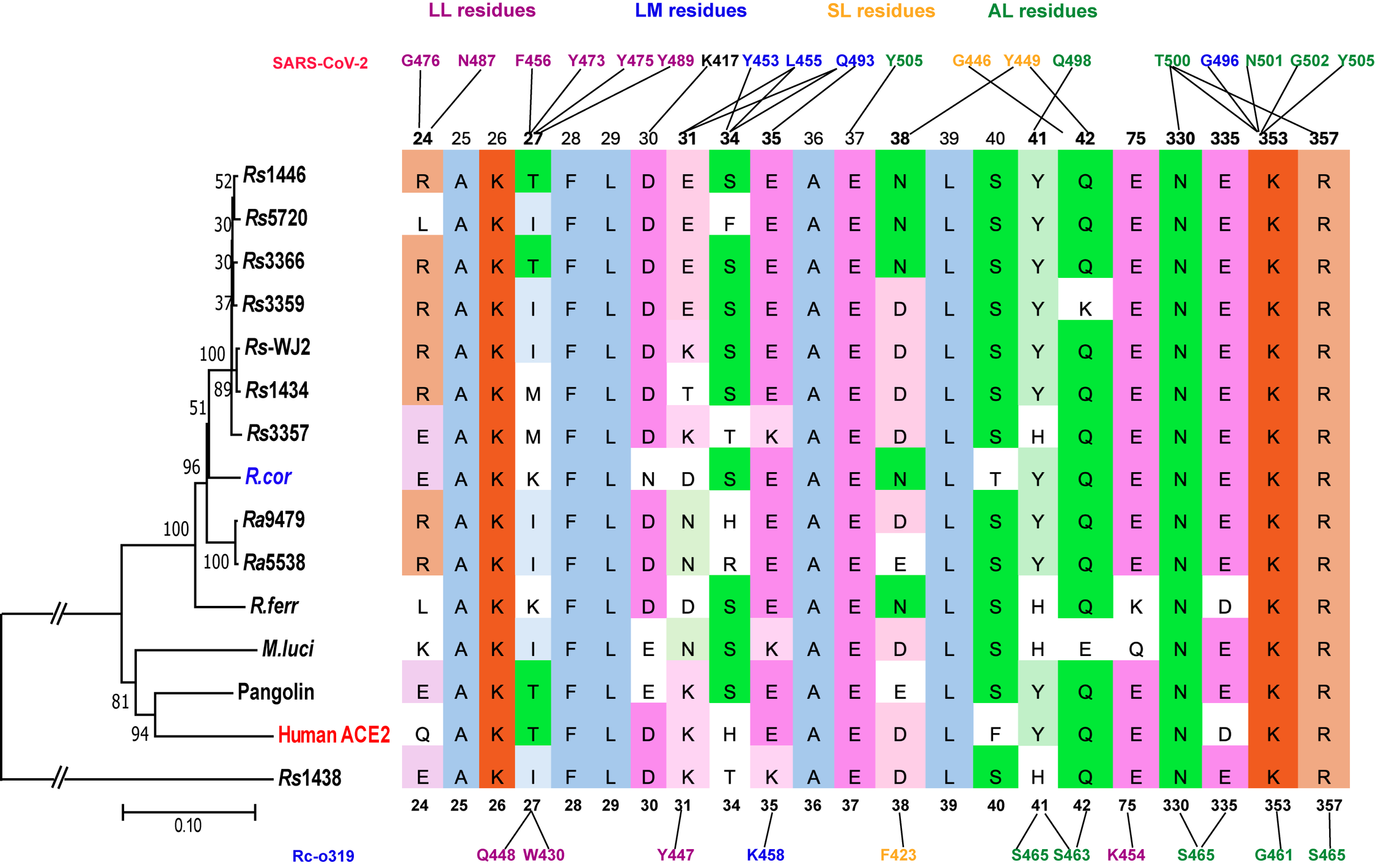

Supplement: S17 Fig — The phylogenetic tree was generated with maximum likelihood analysis. The multi-sequence alignment was analyzed using MAFFT and the key interaction residues of sarbecovirus RBD with ACE2 were identified. ACE2 interacting RBM residues of SARS-CoV-2 and Rc-o319 are shown above and below the ACE2 sequence, respectively. Residues in the large loop (LL), lamella (LM), small loop (SL) and anchor loop (AL) regions are colored in purple, blue, orange, and green. Black lines indicate van der Waals contacts, hydrogen bonds, and salt bridges. (TIF) [file ppat.1014245.s017.tif]

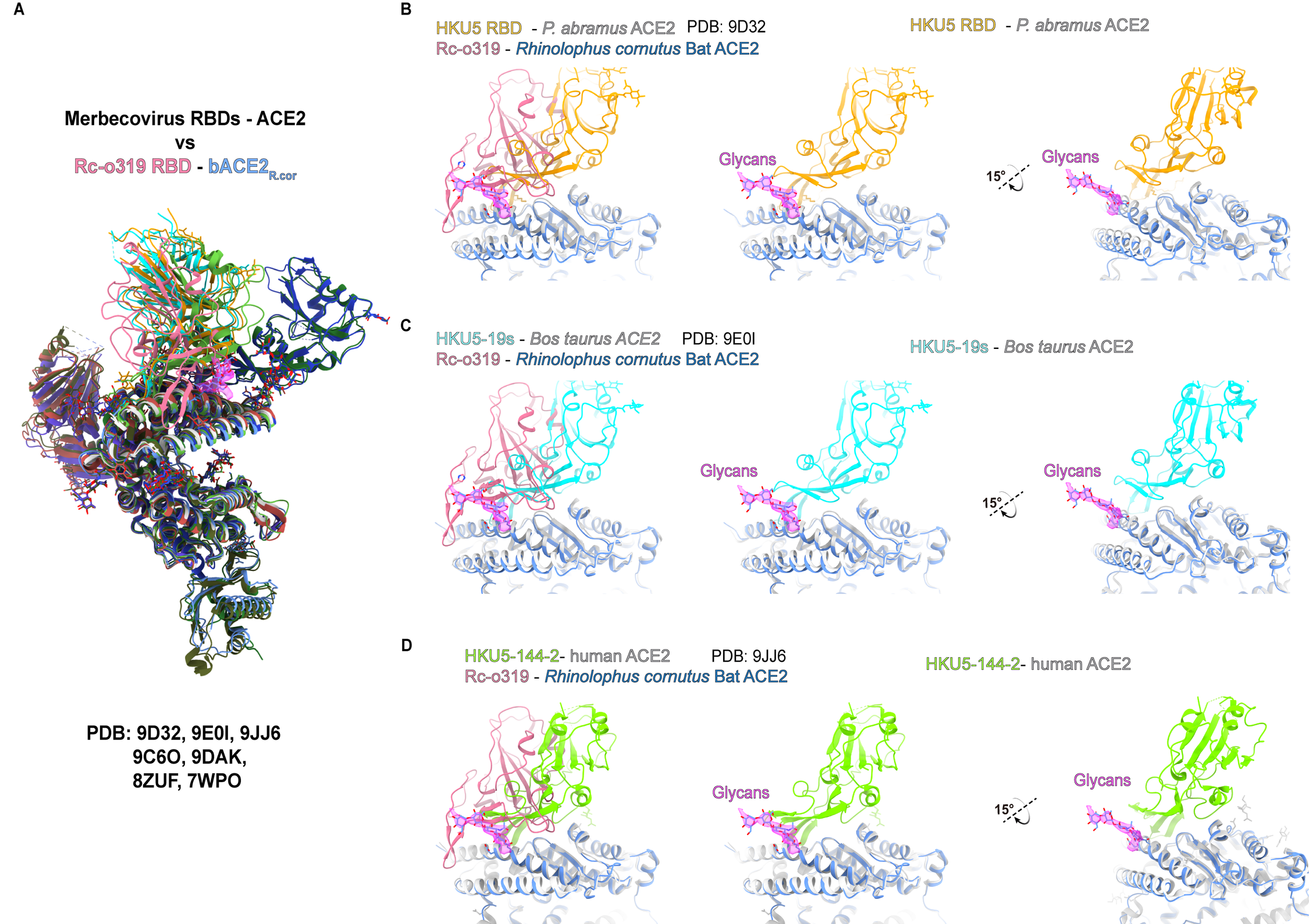

Supplement: S18 Fig — (A) Overview of the structural alignments between the Rc-o319 RBD-bACE2R.cor complex and ACE2-bound merbecovirus RBD complexes, including HKU5 RBD-P.abramus ACE2 (PDB: 9D32), HKU5-19s-Bos taurus ACE2 (PDB: 9E0I), MOW5-22-P. davyi ACE2 (PDB: 9C6O), PnNL2018B-P.nathusii ACE2 (PDB: 9DAK), MOW15-22-P.nat ACE2 (PDB: 8ZUF), NeoCoV-Bat37 ACE2 (PDB: 7WPO), and HKU5-144-2-human ACE2 (PDB: 9JJ6). (B–D) The Asn38-glycan of ACE2 is positioned near the interface between HKU5-like merbecovirus RBDs (HKU5 RBD, HKU5-19s, and HKU5-144-2) and ACE2. (TIF) [file ppat.1014245.s018.tif]
