# Supplementary material for: Structural basis for sarbecovirus Rc-o319 spike adaptation to Rhinolophus cornutus Bat ACE2 and constraints on switching to human ACE2
Source: PLoS Pathog. 2026 May 21;22(5):e1014245. doi: 10.1371/journal.ppat.1014245 (PMC13232947; doi:10.1371/journal.ppat.1014245)
Supplement: S1 Table — (DOCX) [file ppat.1014245.s019.docx]

**S1 Table. Cryo-EM data collection, refinement and validation statistics.**

|  | Rc-o319 S-trimer locked-1 conformation | Rc-o319 S-trimer locked-2 conformation | Rc-o319 RBD:bACE2*_R.cor_* (C2) | Rc-o319 RBD:bACE2*_R.cor_*  (focused map) |  |
| --- | --- | --- | --- | --- | --- |
| **Data collection and processing** |  |  |  |  |  |
|  |  |  |  |  |  |
| Magnification | 130000 X | | 130000 X | |  |
| Voltage (kV) | 300 | | 300 | |  |
| Electron exposure (e^–^/Å^2^) | 50 | | 50 | |  |
| Defocus range (μm) | -0.8 - -2.2 | | -0.8 - -2.0 | |  |
| Pixel size (Å) | 0.93 | | 0.646 | |  |
| Movies (no.) | 6255 | | 39866 | |  |
| Initial particle images (no.) | 732838 | | 1066443 | |  |
| Symmetry imposed | *C3* | | *C2* |  |  |
| Final particle images (no.) | 86699 | 436097 | 493907 | 987814 |  |
| Map resolution (Å) | 2.3 | 2.1 | 2.4 | 2.3 |  |
| FSC threshold | 0.143 | 0.143 | 0.143 | 0.143 |  |
| Map resolution range (Å) | 2.08-4.33 | 2.08-3.21 | 2.00-3.96 | 1.96-3.97 |  |
|  |  |  |  |  |  |
| **Refinement** |  |  |  |  |  |
| Initial model used | 7XU2 | 7XU2 | 8ZY9 | 8ZYA |  |
| Model resolution (Å) | 3.2 | 3.2 | 2.7 | 2.5 |  |
| FSC threshold | 0.5 | 0.5 | 0.5 | 0.5 |  |
| Map sharpening *B* factor (Å^2^) | 58.1 | 63.8 | 92.5 | 86.1 |  |
| Model composition |  |  |  |  |  |
| Non-hydrogen atoms | 26247 | 26002 | 14274 | 7315 |  |
| Protein residues | 3231 | 3182 | 1695 | 868 |  |
| Ligands | 69 | 75 | 26 | 15 |  |
| *B* factors (Å^2^) |  |  |  |  |  |
| Protein | 39.77 | 44.09 | 56.27 | 18.73 |  |
| Ligand | 79.97 | 56.19 | 103.47 | 35.79 |  |
| R.m.s. deviations |  |  |  |  |  |
| Bond lengths (Å) | 0.003 | 0.003 | 0.004 | 0.004 |  |
| Bond angles (°) | 0.575 | 0.652 | 0.781 | 0.698 |  |
|  |  |  |  |  |  |
| **Validation** |  |  |  |  |  |
| MolProbity score | 1.23 | 1.63 | 1.75 | 1.71 |  |
| Clashscore | 2.76 | 3.39 | 5.02 | 4.49 |  |
| Poor rotamers (%) | 0.00 | 0.00 | 0.00 | 0.00 |  |
| Ramachandran plot |  |  |  |  |  |
| Favored (%) | 97.64 | 95.94 | 96.36 | 97.10 |  |
| Allowed (%) | 2.36 | 4.06 | 3.64 | 2.90 |  |
| Disallowed (%) | 0.00 | 0.00 | 0.00 | 0.00 |  |
