# Supplementary material for: Structural basis for sarbecovirus Rc-o319 spike adaptation to Rhinolophus cornutus Bat ACE2 and constraints on switching to human ACE2
Source: PLoS Pathog. 2026 May 21;22(5):e1014245. doi: 10.1371/journal.ppat.1014245 (PMC13232947; doi:10.1371/journal.ppat.1014245)
Supplement: S2 Table — (DOCX) [file ppat.1014245.s020.docx]

**S2 Table.** **Kinetic parameters of different ACE2 orthologs binding to different constructs of wild-type Rc-o319 S-proteins (related to Figure 1).**

| ACE2  ortholog | Rc-o319-6P  S-protein | | |  | Rc-o319  S-RBD-Fc | | |
| --- | --- | --- | --- | --- | --- | --- | --- |
|  | *k*_on_ (M^-1^S^-1^) | *k*_off_ (S^-1^) | *K_D_* (nM) |  | *k*_on_ (M^-1^S^-1^) | *k*_off_ (S^-1^) | *K_D_* (nM) |
| bACE2*_R.cor_* | 9.640x10^3^  (*k*_on1_) | 2.085x10^-2^  (*k*_off1_) | 2163  (*k*_off1_/*k*_on1_) | bACE2*_R.cor_* | 8.523 x 10^4^ (*k*_on_) | 6.468 x 10^-3^  (*k*_off_) | 75.9  (*k*_off_/*k*_on_) |
|  | 3.899x10^4^  (*k*_on2_) | 2.555x10^-4^  (*k*_off2_) | 534.7  (*k*_off1_/*k*_on2_) |  |  |  |  |
|  |  |  | 26.5  (*k*_off2_/*k*_on1_) |  |  |  |  |
|  |  |  | 6.6  (*k*_off2_/*k*_on2_) |  |  |  |  |
|  |  |  |  |  |  |  |  |
| bACE2*_Ra_*_9479_ | - | - | No binding | bACE2*_Ra_*_9479_ | - | - | No binding |
|  |  |  |  |  |  |  |  |
| hACE2 | - | - | No binding | hACE2 | - | - | No binding |
