# Supplementary material for: Structural basis for sarbecovirus Rc-o319 spike adaptation to Rhinolophus cornutus Bat ACE2 and constraints on switching to human ACE2
Source: PLoS Pathog. 2026 May 21;22(5):e1014245. doi: 10.1371/journal.ppat.1014245 (PMC13232947; doi:10.1371/journal.ppat.1014245)
Supplement: S3 Table — (DOCX) [file ppat.1014245.s021.docx]

**S3 Table. Kinetic parameters of different Rc-o319 RBD-Fc variants binding to bACE2*_R.cor_* or hACE2 (related to Figure 4).**

| Rc-o319 RBD variants | bACE2*_R.cor_* | | | | | | Rc-o319 RBD variants | | bACE2*_R.cor_* | | | | | |
| --- | --- | --- | --- | --- | --- | --- | --- | --- | --- | --- | --- | --- | --- | --- |
|  | *k*_on_ (M^-1^S^-1^) | | *k*_off_ (S^-1^) | | *K_D_* (nM) | |  | | *k*_on_ (M^-1^S^-1^) | | *k*_off_ (S^-1^) | | *K_D_* (nM) | |
| WT | 8.523 x 10^4^  (*k*_on_) | | 6.468 x 10^-3^  (*k*_off_) | | 75.9  (*k*_off_/*k*_on_) | | BL | | - | | - | | No Binding | |
| LM  (K458Q) | 1.612 x 10^4^  (*k*_on_) | | 4.474 x 10^-2^  (*k*_off_) | | 2775  (*k*_off_/*k*_on_) | | AL  (S465T  A466N H470Y) | | 6.023 x 10^4^  (*k*_on_) | | 3.656 x 10^-3^  (*k*_off_) | | 60.7  (*k*_off_/*k*_on_) | |
| SL  (F423Y) | 8.789 x 10^4^  (*k*_on_) | | 7.532 x10^-3^  (*k*_off_) | | 85.7  (*k*_off_/*k*_on_) | | BL+  +LM+AL+SL | | - | | - | | No Binding | |
| BL+LM+AL+  SL+RBM-loop | - | | - | | No Binding | | Rc-o319-RBD-RBM_SARS2_ | | - | | - | | No Binding | |
| BL+LM | - | | - | | No binding | | BL+RBM-loop | | 8.215 x 10^3^  (*k*_on_) | | 3.970 x 10^-2^  (*k*_off_) | | 483  (*k*_off_/*k*_on_) | |
| BL+LM+RBM-loop | - | | - | | No binding | | SARS-CoV-2 | | - | | - | | No binding | |
| Rc-o319-RBD variants | | hACE2 | | | | | | Rc-o319-RBD variants | | hACE2 | | | | |
|  | | *k*_on_ (M^-1^S^-1^) | | *k*_off_ (S^-1^) | | *K*_D_ (nM) | |  | | *k*_on_ (M^-1^S^-1^) | | *k*_off_ (S^-1^) | | *K*  (nM) |
| WT | | - | | - | | No Binding | | BL | | - | | - | | No Binding |
| LM | | - | | - | | No Binding | | AL | | - | | - | | No Binding |
| SL | | - | | - | | No Binding | | BL+ LM  +AL+SL | | - | | - | | No Binding |
| BL+LM+  AL+SL +  RBM-Loop | | 2.930 x 10^4^  (*k*_on_) | | 9.286 x 10^-4^  *(k*_off_) | | 31.7  (*k*_off_/*k*_on_) | | Rc-o319-RBD-SARS2_RBM_ | | 3.520 x 10^4^  (*k*_on_) | | 4.331 x 10^-4^  (*k*_off_) | | 12.3  (*k*_off_/*k*_on_) |
| BL+LM | | - | | - | | No binding | | BL+RBM-loop | | 2.027 x 10^4^  (*k*_on_) | | 3.218x 10^-2^  (*k*_off_) | | 1588  (*k*_off_/*k*_on_) |
| BL+LM+RBM-loop | | 6.594 x 10^3^  (*k*_on_) | | 9.155x 10^-3^  (*k*_off_) | | 1388.4  (*k*_off_/*k*_on_) | | SARS-CoV-2 | | 7.277 x 10^3^  (*k*_on_) | | 4.783 x 10^-5^  (*k*_off_) | | 0.065  (*k*_off_/*k*_on_) |
