# Supplementary material for: Structural basis for sarbecovirus Rc-o319 spike adaptation to Rhinolophus cornutus Bat ACE2 and constraints on switching to human ACE2
Source: PLoS Pathog. 2026 May 21;22(5):e1014245. doi: 10.1371/journal.ppat.1014245 (PMC13232947; doi:10.1371/journal.ppat.1014245)
Supplement: S4 Table — (DOCX) [file ppat.1014245.s022.docx]

**S4 Table. Kinetic parameters of different sarbecovirus RBD-Fc proteins and their variants binding to bACE2*_R.cor_* (related to Fig. S9).**

| Rc-o319 RBD variants | bACE2*_R.cor_* | | | | Rc-o319 RBD variants | bACE2*_R.cor_* | | | | | |  |
| --- | --- | --- | --- | --- | --- | --- | --- | --- | --- | --- | --- | --- |
|  | *k*_on_ (M^-1^S^-1^) | *k*_off_ (S^-1^) | | *K_D_* (nM) |  | *k*_on_ (M^-1^S^-1^) | *k*_off_ (S^-1^) | | | *K_D_* (nM) | |  |
| WT | 6.863 x 10^4^  (*k*_on_) | 1.654 x 10^-2^  (*k*_off_) | | 241.0  (*k*_off_/*k*_on_) | K454Y | 1.476 x 10^5^  (*k*_on_) | | 1.803 x 10^-1^  (*k*_off_) | | | 1221.5  (*k*_off_/*k*_on_) | |
|  |  |  | |  |  |  | |  | | |  | |
| LM  (K458Q) | 1.612 x 10^4^  (*k*_on_) | 4.474 x 10^-2^  (*k*_off_) | | 2775  (*k*_off_/*k*_on_) | LM  (K458A) | 6.177 x 10^4^  (*k*_on_) | | 1.483 x 10^-1^  (*k*_off_) | | | Weak binding | |
| Sarbecovirus-RBD variants | bACE2*_R.cor_* | | | | Sarbecovirus-RBD variants | bACE2*_R.cor_* | | | | | |  |
|  | *k*_on_ (M^-1^S^-1^) | | *k*_off_ (S^-1^) | *K_D_* (nM) |  | *k*_on_ (M^-1^S^-1^) | *k*_off_ (S^-1^) | | *K_D_* (nM) | | |  |
| BANAL-20-52 | 9.891 x 10^4^  (*k*_on_) | 6.288 x 10^-2^  (*k*_off_) | | Weak binding  (*k*_off_/*k*_on_) | BANAL-20-236 | 2.731 x 10^4^  (*k*_on_) | 3.580 x10^-4^  (*k*_off_) | | 13.1  (*k*_off_/*k*_on_) | | |  |
|  |  |  | |  |  |  |  | |  | | |  |
| SARS1-N479K | - | - | | Weak binding | SARS2-Q493K | 3.191 x 10^4^  (*k*_on_) | 2.435 x 10^-2^  (*k*_off_) | | 763.0  (*k*_off_/*k*_on_) | | |  |
