# Supplementary material for: Structural basis for sarbecovirus Rc-o319 spike adaptation to Rhinolophus cornutus Bat ACE2 and constraints on switching to human ACE2
Source: PLoS Pathog. 2026 May 21;22(5):e1014245. doi: 10.1371/journal.ppat.1014245 (PMC13232947; doi:10.1371/journal.ppat.1014245)
Supplement: S5 Table — (DOCX) [file ppat.1014245.s023.docx]

**S5 Table. Kinetic parameters of hACE2, bACE2*_R.cor_* or bACE2*_R.cor_*-ΔGLC_38_ binding to different Rc-o319 S-trimers (related to Fig. S10).**

| Spike | bACE2*_R.cor_*-WT | | | hACE2 | | |
| --- | --- | --- | --- | --- | --- | --- |
|  | *k*_on_ (M^-1^S^-1^) | *k*_off_ (S^-1^) | *K_D_* (nM) | *k*_on_ (M^-1^S^-1^) | *k*_off_ (S^-1^) | *K_D_* (nM) |
| Rc-o319 | 7.746 x 10^3^  (*k*_on1_) | 1.467 x 10^-2^(*k*_off1_) | 1894  (k_off1_/k_on1_) | - | - | No Binding |
|  | 1.045 x 10^5^  (*k*_on2_) | 4.303 x 10^-4^  (*k*_off2_) | 140.4  (*k*_off1_/*k*_on2_) |  |  |  |
|  |  |  | 55.6  (*k*_off2_/*k*_on1_) |  |  |  |
|  |  |  | 4.1  (*k*_off2_/*k*_on2_) |  |  |  |
|  |  |  |  |  |  |  |
|  | *k*_on_ (M^-1^S^-1^) | *k*_off_ (S^-1^) | *K_D_* (nM) | *k*_on_ (M^-1^S^-1^) | *k*_off_ (S^-1^) | *K_D_* (nM) |
| BL | - | - | No Binding | 2.966 x 10^3^  (*k*_on1_) | 9.203x 10^-2^  (*k*_off1_) | 31024  (k_off1_/k_on1_) |
|  |  |  |  | 7.408x 10^4^  (*k*_on2_) | 1.193 x 10^-3^  (*k*_off2_) | 1242.3  (k_off1_/k_on2_) |
|  |  |  |  |  |  | 402.0  (k_off2_/k_on1_) |
|  |  |  |  |  |  | 16.1  (k_off2_/k_on2_) |
|  |  |  |  |  |  |  |
|  | *k*_on_ (M^-1^S^-1^) | *k*_off_ (S^-1^) | *K_D_* (nM) | *k*_on_ (M^-1^S^-1^) | *k*_off_ (S^-1^) | *K_D_* (nM) |
| AL | 4.557 x 10^3^  (*k*_on1_) | 1.333 x 10^-2^  (*k*_off1_) | 2925.0  (*k*_off1_/*k*_on1_) | - | - | No Binding |
|  | 4.437 x 10^4^  (*k*_on2_) | 2.223 x 10^-4^  (*k*_off2_) | 300.4  (*k*_off1_/*k*_on2_) |  |  |  |
|  |  |  | 48.8  (*k*_off2_/*k*_on1_) |  |  |  |
|  |  |  | 5.0  (*k*_off2_/*k*_on2_) |  |  |  |
|  |  |  |  |  |  |  |
|  | *k*_on_ (M^-1^S^-1^) | *k*_off_ (S^-1^) | *K_D_* (nM) | *k*_on_ (M^-1^S^-1^) | *k*_off_ (S^-1^) | *K_D_* (nM) |
| BL+LM+AL+SL | - | - | No Binding | 5.649 x 10^3^  (*k*_on1_) | 5.611 x 10^-2^  (*k*_off1_) | 9933.5  (*k*_off1_/*k*_on1_) |
|  |  |  |  | 1.621 x 10^5^  (*k*_on2_) | 6.281 x 10^-4^  (*k*_off2_) | 346.2  (*k*_off1_/*k*_on2_) |
|  |  |  |  |  |  | 111.2  (*k*_off2_/*k*_on1_) |
|  |  |  |  |  |  | 3.9  (*k*_off2_/*k*_on2_) |
|  |  |  |  |  |  |  |
|  | *k*_on_ (M^-1^S^-1^) | *k*_off_ (S^-1^) | *K_D_* (nM) | *k*_on_ (M^-1^S^-1^) | *k*_off_ (S^-1^) | *K_D_* (nM) |
| BL+LM+AL+SL  +RBM-loop | - | - | No Binding | 1.948 x 10^3^  (*k*_on1_) | 3.316 x 10^-4^  (*k*_off1_) | 170.3  (*k*_off1_/*k*_on1_) |
|  |  |  |  | 1.211 x 10^4^  (*k*_on2_) | < 1 x 10^-7^  (*k*_off2_) | 27.4  (*k*_off1_/*k*_on2_) |
|  |  |  |  |  |  | < 0.01  (*k*_off2_/*k*_on1_) |
|  |  |  |  |  |  | < 0.01  (*k*_off2_/*k*_on2_) |
| ACE2 | BL | | | BL+LM+AL+SL  +RBM-loop | | |
|  | *k*_on_ (M^-1^S^-1^) | *k*_off_ (S^-1^) | *K_D_* (nM) | *k*_on_ (M^-1^S^-1^) | *k*_off_ (S^-1^) | *K_D_* (nM) |
| bACE2*_R.cor_*-ΔGLC_38_ | 8.317 x 10^3^  (*k*_on1_) | 5.669 x 10^-2^  (*k*_off1_) | 6816.2  (*k*_off1_/*k*_on1_) | 4.774 x 10^3^  (*k*_on1_) | 9.353 x 10^-2^  (*k*_off1_) | 19593  (*k*_off1_/*k*_on1_) |
|  | 9.335 x 10^7^  (*k*_on2_) | 1.110 x 10^-3^  (*k*_off2_) | 133.4  (*k*_off1_/*k*_on2_) | 2.253 x 10^5^  (*k*_on2_) | < 1 x 10^-7^  (*k*_off2_) | 415.2  (*k*_off1_/k_on2_) |
|  |  |  | 0.6  (*k*_off2_/*k*_on1_) |  |  | <0.01  (*k*_off2_/*k*_on1_) |
|  |  |  | 0.01  (*k*_off2_/*k*_on2_) |  |  | <0.01  (*k*_off2_/*k*_on2_) |
