# Supplementary material for: Structural basis for sarbecovirus Rc-o319 spike adaptation to Rhinolophus cornutus Bat ACE2 and constraints on switching to human ACE2
Source: PLoS Pathog. 2026 May 21;22(5):e1014245. doi: 10.1371/journal.ppat.1014245 (PMC13232947; doi:10.1371/journal.ppat.1014245)
Supplement: S6 Table — (DOCX) [file ppat.1014245.s024.docx]

**S6 Table. Kinetic parameters of bACE2*_R.cor_* or bACE2*_R.cor_*-ΔGLC_38_ binding to different sarbecovirus S-trimers (related to Fig. S11A-H).**

| Spike | bACE2*_R.cor_*-WT | | | bACE2*_R.cor_*-ΔGLC_38_ | | |
| --- | --- | --- | --- | --- | --- | --- |
|  | *k*_on_ (M^-1^S^-1^) | *k*_off_ (S^-1^) | *K_D_* (nM) | *k*_on_ (M^-1^S^-1^) | *k*_off_ (S^-1^) | *K_D_* (nM) |
| Rc-o319 | 3.131 x 10^3^  (*k*_on1_) | 1.048 x 10^-2^  (*k*_off1_) | 3347.5  (*k*_off1_/*k*_on1_) | 5.372 x 10^3^  (*k*_on1_) | 2.347 x 10^-3^  (*k*_off1_) | 436.9  (*k*_off1_/*k*_on1_) |
|  | 3.503 x 10^4^  (*k*_on2_) | 3.474 x 10^-4^  (*k*_off2_) | 299.2  (*k*_off1_/*k*_on2_) | 2.585 x 10^4^  (*k*_on2_) | < 1 x 10^-7^  (*k*_off2_) | 90.8  (*k*_off1_/k_on2_) |
|  |  |  | 111.0  (*k*_off2_/*k*_on1_) |  |  | <0.0186  (*k*_off2_/*k*_on1_) |
|  |  |  | 9.9  (*k*_off2_/*k*_on2_) |  |  | <0.0038  (*k*_off2_/*k*_on2_) |
|  |  |  |  |  |  |  |
|  | *k*_on_ (M^-1^S^-1^) | *k*_off_ (S^-1^) | *K_D_* (nM) | *k*_on_ (M^-1^S^-1^) | *k*_off_ (S^-1^) | *K_D_* (nM) |
| BANAL-20-52 | - | - | No Binding | 3.049 x 10^3^  (*k*_on1_) | 6.073x 10^-5^  (*k*_off1_) | 19.9  (k_off1_/k_on1_) |
|  |  |  |  | 3.109x 10^3^  (*k*_on2_) | < 1 x 10^-7^  (*k*_off2_) | 19.6  (k_off1_/k_on2_) |
|  |  |  |  |  |  | <0.033  (k_off2_/k_on1_) |
|  |  |  |  |  |  | <0.032  (k_off2_/k_on2_) |
|  |  |  |  |  |  |  |
| BANAL-20-236 | 6.635 x 10^3^  (*k*_on1_) | 2.194 x 10^-4^  (*k*_off1_) | 33.1  (*k*_off1_/*k*_on1_) | 6.800 x 10^3^  (*k*_on1_) | 2.234 x 10^-4^  (*k*_off1_) | 32.9  (*k*_off1_/*k*_on1_) |
|  | 7.280 x 10^3^  (*k*_on2_) | <1 x 10^-7^  (*k*_off2_) | 30.1  (*k*_off1_/*k*_on2_) | 3.320 x 10^4^  (*k*_on2_) | <1 x 10^-7^  (*k*_off2_) | 6.73  (*k*_off1_/*k*_on2_) |
|  |  |  | <0.015  (*k*_off2_/*k*_on1_) |  |  | <0.015  (*k*_off2_/*k*_on1_) |
|  |  |  | <0.014  (*k*_off2_/*k*_on2_) |  |  | <0.003  (*k*_off2_/*k*_on2_) |
|  |  |  |  |  |  |  |
|  | *k*_on_ (M^-1^S^-1^) | *k*_off_ (S^-1^) | *K_D_* (nM) | *k*_on_ (M^-1^S^-1^) | *k*_off_ (S^-1^) | *K_D_* (nM) |
| BtKY72 | - | - | No Binding | 5.637 x 10^3^  (*k*_on1_) | 1.016 x 10^-2^  (*k*_off1_) | 1802.7  (*k*_off1_/*k*_on1_) |
|  |  |  |  | 2.747 x 10^4^  (*k*_on2_) | 3.250 x 10^-4^  (*k*_off2_) | 370.0  (*k*_off1_/*k*_on2_) |
|  |  |  |  |  |  | 57.7  (*k*_off2_/*k*_on1_) |
|  |  |  |  |  |  | 11.8  (*k*_off2_/*k*_on2_) |
