# Supplementary material for: Structural basis for sarbecovirus Rc-o319 spike adaptation to Rhinolophus cornutus Bat ACE2 and constraints on switching to human ACE2
Source: PLoS Pathog. 2026 May 21;22(5):e1014245. doi: 10.1371/journal.ppat.1014245 (PMC13232947; doi:10.1371/journal.ppat.1014245)
Supplement: S7 Table — (DOCX) [file ppat.1014245.s025.docx]

**S7 Table. Kinetic parameters of bACE2_Ra9479_-GLC_38_ or bACE2_Ra9479_ binding to different sarbecovirus S-trimers (related to S11J-L Fig).**

| Spike | bACE2_Ra9479_-GLC_38_ | | | bACE2_Ra9479_-WT | | |
| --- | --- | --- | --- | --- | --- | --- |
|  | *k*_on_ (M^-1^S^-1^) | *k*_off_ (S^-1^) | *K_D_* (nM) | *k*_on_ (M^-1^S^-1^) | *k*_off_ (S^-1^) | *K_D_* (nM) |
| SARS-CoV-1 | 3.995 x 10^3^  (*k*_on1_) | 1.886 x 10^-2^  (*k*_off1_) | 4722.4  (*k*_off1_/*k*_on1_) | 4.073 x 10^3^  (*k*_on1_) | 2.290 x 10^-4^  (*k*_off1_) | 56.2  (*k*_off1_/*k*_on1_) |
|  | 7.279 x 10^3^  (*k*_on2_) | 1.152 x 10^-4^  (*k*_off2_) | 2591.6  (*k*_off1_/*k*_on2_) | 3.079 x 10^4^  (*k*_on2_) | 3.784 x 10^-7^  (*k*_off2_) | 7.4  (*k*_off1_/k_on2_) |
|  |  |  | 28.8  (*k*_off2_/*k*_on1_) |  |  | 0.093  (*k*_off2_/*k*_on1_) |
|  |  |  | 15.8  (*k*_off2_/*k*_on2_) |  |  | 0.012  (*k*_off2_/*k*_on2_) |
|  |  |  |  |  |  |  |
|  | *k*_on_ (M^-1^S^-1^) | *k*_off_ (S^-1^) | *K_D_* (nM) | *k*_on_ (M^-1^S^-1^) | *k*_off_ (S^-1^) | *K_D_* (nM) |
| BtKY72 | 8.959 x 10^3^  (*k*_on1_) | 1.234 x 10^-2^  (*k*_off1_) | 1377.8  (*k*_off1_/*k*_on1_) | 4.134 x 10^3^  (*k*_on1_) | 2.182x 10^-3^  (*k*_off1_) | 528.0  (k_off1_/k_on1_) |
|  | 9.159 x 10^4^  (*k*_on2_) | 1.909 x 10^-4^  (*k*_off2_) | 134.7  (*k*_off1_/*k*_on2_) | 5.934x 10^4^  (*k*_on2_) | < 1 x 10^-7^  (*k*_off2_) | 36.8  (k_off1_/k_on2_) |
|  |  |  | 21.3  (*k*_off2_/*k*_on1_) |  |  | 0.012  (k_off2_/k_on1_) |
|  |  |  | 2.1  (*k*_off2_/*k*_on2_) |  |  | <0.01  (k_off2_/k_on2_) |
| RBD | bACE2*_R.cor_*-WT | | |  |  |  |
|  | *k*_on_ (M^-1^S^-1^) | *k*_off_ (S^-1^) | *K_D_* (nM) |  |  |  |
| BANAL-20-236 | 1.149 x 10^4^  (*k*_on_) | 1.126 x 10^-2^  (*k*_off_) | 980.5  (*k*_off_/*k*_on_) |  |  |  |
