# Supplementary material for: Structural basis for sarbecovirus Rc-o319 spike adaptation to Rhinolophus cornutus Bat ACE2 and constraints on switching to human ACE2
Source: PLoS Pathog. 2026 May 21;22(5):e1014245. doi: 10.1371/journal.ppat.1014245 (PMC13232947; doi:10.1371/journal.ppat.1014245)
Supplement: S8 Table — (DOCX) [file ppat.1014245.s026.docx]

**S8 Table. Kinetic parameters of different variants of SARS-CoV-2-RBD-Fc protein binding to bACE2*_R.cor_* or hACE2 (related to Fig. S12).**

| SARS-CoV-2-RBD-Fc  variants | bACE2*_R.cor_* | | | hACE2 | | | | |  |
| --- | --- | --- | --- | --- | --- | --- | --- | --- | --- |
|  | *k*_on_ (M^-1^S^-1^) | *k*_off_ (S^-1^) | *K_D_* (nM) | *k*_on_ (M^-1^S^-1^) | *k*_off_ (S^-1^) | | *K_D_* (nM) | |  |
| BL_Rc-o319_ | - | - | No binding | - | | - | | No binding | |
| BL_Rc-o319_ + RBM-loop_Rc-o319_ | - | - | No binding | - | | - | | No binding | |
|  |  |  |  |  | |  | |  | |
| BL_Rc-o319_ + LM_Rc-o319_ | - | - | Weak binding | - | | - | | No binding | |
|  |  |  |  |  | |  | |  | |
| RBM_Rc-o319_ | 8.133x 10^4^  (*k*_on_) | 6.596 x 10^-3^  (*k*_off_) | 81.1  (*k*_off_/*k*_on_) | - | | - | | No binding | |
