# Supplementary material for: Structural basis for sarbecovirus Rc-o319 spike adaptation to Rhinolophus cornutus Bat ACE2 and constraints on switching to human ACE2
Source: PLoS Pathog. 2026 May 21;22(5):e1014245. doi: 10.1371/journal.ppat.1014245 (PMC13232947; doi:10.1371/journal.ppat.1014245)
Supplement: S9 Table — (DOCX) [file ppat.1014245.s027.docx]

**S9 Table. Kinetic parameters of different Rc-o319 RBD-Fc variants binding to bACE2*_R.cor_* (related to Fig. S14).**

| Rc-o319 RBD variants | bACE2*_R.cor_* | | | Rc-o319 RBD variants | bACE2*_R.cor_* | | |
| --- | --- | --- | --- | --- | --- | --- | --- |
|  | *k*_on_ (M^-1^S^-1^) | *k*_off_ (S^-1^) | *K_D_* (nM) |  | *k*_on_ (M^-1^S^-1^) | *k*_off_ (S^-1^) | *K_D_* (nM) |
| WT | 2.385 x 10^4^  (*k*_on_) | 2.922 x 10^-3^  (*k*_off_) | 122.5  (*k*_off_/*k*_on_) | S465T | 3.510 x 10^4^  (*k*_on_) | 1.701 x 10^-3^  (*k*_off_) | 48.5  (*k*_off_/*k*_on_) |
| A466N | 1.147 x 10^5^  (*k*_on_) | 4.568 x 10^-2^  (*k*_off_) | 398.4  (*k*_off_/*k*_on_) | H470Y | 4.621 x 10^4^  (*k*_on_) | 2.987 x 10^-3^  (*k*_off_) | 64.6  (*k*_off_/*k*_on_) |
| S465T  +A466N | 3.454 x 10^4^  (*k*_on_) | 1.077 x10^-3^  (*k*_off_) | 31.2  (*k*_off_/*k*_on_) | S465T+  A466N+ H470Y | 4.429 x 10^4^  (*k*_on_) | 2.498 x10^-3^  (*k*_off_) | 56.4  (*k*_off_/*k*_on_) |
