# Supplementary material for: Structural basis for sarbecovirus Rc-o319 spike adaptation to Rhinolophus cornutus Bat ACE2 and constraints on switching to human ACE2
Source: PLoS Pathog. 2026 May 21;22(5):e1014245. doi: 10.1371/journal.ppat.1014245 (PMC13232947; doi:10.1371/journal.ppat.1014245)
Supplement: S10 Table — (DOCX) [file ppat.1014245.s028.docx]

**S10 Table. Key ACE2 residues in the ACE2-RBD interface of the orthologs tested in the cell-cell fusion assay (related to Figure 1 and supplymentary Fig.17).**

| **Species** | **Isolate #** | **Genbank** | **ACE2-RBD Contact Residues** | | | | | | | | | | | | | | | |
| --- | --- | --- | --- | --- | --- | --- | --- | --- | --- | --- | --- | --- | --- | --- | --- | --- | --- | --- |
|  |  |  | **24** | **27** | **30** | **31** | **34** | **35** | **38** | **40** | **41** | **42** | **45** | **82** | **83** | **330** | **353** | **355** |
| ***R. cornutus*** | *R.cor* | BCG67443.1 | E | **K** | N | D | S | E | **N** | T | Y | Q | L | N | Y | N | K | D |
| ***R. sinicus*** | WJ2 | QMQ39202.1 | R | **I** | D | K | S | E | D | S | Y | Q | L | N | Y | N | K | D |
| ***R. sinicus*** | 1446 | MT394194.1 | R | **T** | D | E | S | E | N | S | Y | Q | L | N | Y | N | K | D |
| ***R. sinicus*** | 1434 | QMQ39216.1 | R | **M** | D | T | S | E | D | S | Y | Q | L | N | Y | N | K | D |
| ***R. sinicus*** | 1438 | MT394184.1 | E | **I** | D | K | T | K | D | S | H | Q | L | N | Y | N | K | D |
| ***R. sinicus*** | 3357 | AGZ48803.1 | E | **M** | D | K | T | K | D | S | H | Q | L | N | Y | N | K | D |
| ***R. sinicus*** | 3366 | QMQ39215.1 | R | **T** | D | E | S | E | **N** | S | Y | Q | L | N | Y | N | K | D |
| ***R. sinicus*** | 3359 | QMQ39211.1 | R | **I** | D | E | S | E | D | S | Y | K | L | N | Y | N | K | D |
| ***R. sinicus*** | 5720 | MT394182.1 | L | **I** | D | E | F | E | **N** | S | Y | Q | L | N | Y | N | K | D |
| ***R. affinis*** | 9479 | MT394208.1 | R | **I** | D | N | H | E | D | S | Y | Q | L | N | Y | N | K | D |
| ***R. affinis*** | 787 | MT394203.1 | R | **I** | D | N | R | E | E | S | Y | Q | L | N | Y | N | K | D |
| ***R. ferrumequinum*** | *R.ferr* | BAH02663.1 | L | **K** | D | D | S | E | **N** | S | H | Q | L | N | F | N | K | D |
| ***Myotis lucifugus*** | *M.luci* | XP_023609438.1 | K | **I** | E | N | S | K | D | S | H | E | L | N | Y | N | K | D |
| **Pangolin** | - | XP_017505752.1 | E | **T** | E | K | S | E | E | S | Y | Q | L | N | Y | N | K | D |
| **Human** | - | BAB40370.1 | Q | **T** | D | K | H | E | D | F | Y | Q | L | M | Y | N | K | D |
