# Supplementary material for: Structural basis for sarbecovirus Rc-o319 spike adaptation to Rhinolophus cornutus Bat ACE2 and constraints on switching to human ACE2
Source: PLoS Pathog. 2026 May 21;22(5):e1014245. doi: 10.1371/journal.ppat.1014245 (PMC13232947; doi:10.1371/journal.ppat.1014245)
Supplement: Attachment — Submitted filename: Response to reviewers-20260210.docx [file ppat.1014245.s030.docx]

Response to reviewers:

Reviewer #1:

1. How were the two conformations (lock-1 and lock-2) of the Rc-o319 spike identified through 3D classification during cryo-EM data processing? Given their high similarity, is there any possibility of artificial factors influencing their identification during the classification process? Could you provide specific cryo-EM density evidence to support the distinction between these two conformations?

**Response:**

We thank the reviewer for this comment. We have now added more details in the *Methods* Section regarding cryo-EM data processing to clarify how we identified the two conformations. We further added additional figures (Fig. S4F-G) to show densities distinguishing the two distinct conformations.

During cryo-EM data processing, *ab initio* 3D classification was performed in cryoSPARC with the resolution limit restricted to 10.0 Å to minimize overfitting while resolving large-scale conformational differences. Eight initial classes were obtained from this procedure. Three good classes were subsequently subjected to independent non-uniform refinement. The resulting refined maps were then rigid-body aligned and compared using pairwise map-map correlation analysis to quantitatively assess structural similarity among the classes.

The correlation coefficient between class 1 and class 2 was 0.98, indicating that these two classes represent essentially the same conformation. In contrast, the correlation between class 1 and class 4 was substantially lower (0.8787), supporting class 4 being a distinct conformation. Based on this analysis, particles from classes 1 and 2 were grouped and further refined, whereas particles from class 4 were refined independently.

After final refinement, the two particle subsets yielded two S-trimer structures adopting distinct conformations. Based on our previous definitions, the structure refined from class 4 particles corresponds to the locked-1 conformation, in which domain D contains a large disordered loop. In contrast, in the structure refined from class 1 and class 2 particles, domain D is fully ordered, with the region corresponding to the disordered loop in the locked-1 conformation refolding into two short α-helices (Fig. 1I), consistent with the previously defined locked-2 conformation.

We added additional details in the *Methods* section to clarify our classification procedure, specifying that the resolution was limited to 10.0 Å in the initial classification step to avoid artifacts.

Lines 965-974 of the track-changes MS *Methods* Section:

“For Rc-o319 S-trimer, *ab initio* 3D classification was performed with the resolution limited to 10.0 Å to minimize overfitting while resolving large-scale conformational differences. Eight initial classes were obtained from this procedure. Three well-resolved classes were subsequently subjected to independent non-uniform refinement. The resulting refined maps were then rigid-body aligned and compared using pairwise map-map correlation analysis to assess structural similarity among the classes. Classes yielding similar structures were combined and subjected to final refinement, resulting in two Rc-o319 S-trimer structures adopting distinct conformations.”

We further added cryo-EM density maps highlighting the distinguishing features of domain D in the two conformations, which are shown in Supplementary Figures 4F and 4G (shown below).

In addition, we have added a brief description about locked-1 and locked-2 conformation maps in the main text (lines176-178; 203-206).

Lines 176-178 in the track-changes MS

“Detailed structural analysis reveals tight trimer packing for both conformations. Based on their structural features, the two conformations differ primarily at domain D (**Fig. 2A and B, Fig. S3, S4F** and **G**) and have been …”

Lines 203-206 in the track-changes MS

“Differently, the disordered Domain D loop in the locked-1 conformation refolds and forms a large ordered helical structure, packing against the core of the Domain D and the junction between Domains C and D with non-interupted cryo-EM density (**Fig. 1F** **and** **Fig. S4G**) (25, 40).”

2. In Figures 4B, F, and G, the experimental results regarding receptor binding ability and cell fusion efficiency appear inconsistent. Since strong receptor binding is generally considered a critical step for coronavirus entry, could you clarify the reasoning behind this discrepancy? Additionally, the description of these results in the text could be further elaborated to enhance clarity and understanding.

**Response:**

We thank the reviewer for this important comment, we agree that receptor binding is indeed a critical step in coronavirus entry. However, we believe that the apparent discrepancies between receptor-binding signals measured by *in vitro* biophysical assays (BLI) and functional readouts from cell-cell fusion or pseudovirus entry assays (fluorescence or chemiluminescence) primarily reflect differences in assay sensitivity and assay conditions.

In biophysical BLI assays, relative low-valency analytes (monomeric RBD, dimeric ACE2, or trimeric S-trimer) were used, thereby restricting avidity-mediated binding amplification and limiting sensitivity to weak interactions. In contrast, cell-cell fusion and pseudovirus entry assays (Figs. 4, 5, and S16) involve highly multivalent interactions between multiple surface-displayed S-trimers and ACE2 molecules, resulting in substantially enhanced avidity and sensitivity.

In Figures 4B, F, and G, no detectable binding signal was observed by BLI measuring dimeric ACE2 (with divalent avidity) binding to immobilized dimeric RBD-Fc, whereas the corresponding cell-cell fusion assays showed clear fusion activity. In the revised manuscript, we purified Rc-o319 S-trimers carrying the BL, BL+LM+AL+SL, and BL+LM+AL+SL+RBM-loop changes and assessed their binding to hACE2 and bACE2*_R.cor_* by BLI, with the aim of increasing binding valency from divalent to trivalent to enhance signal detection. For hACE2, clear BLI signals were observed for the BL and BL+LM+AL+SL S-trimer variants (**Fig. S10B** and **10D,** the first panels). These results indicate that increasing binding valency resulted in avidity amplified binding and detectable signals.

However, for bACE2*_R.cor_*, binding by the BL and BL+LM+AL+SL+RBM-loop variant S-trimers was still not detectable. As we found that removal of the Asn38-glycan (ΔGLC_38_) from bACE2*_R.cor_* can enhance S-trimer binding. Therefore, we tested binding of the BL and BL+ LM+AL+SL+RBM-loop variant S-trimers to bACE2*_R.cor_*-ΔGLC_38_, which resulted in weak but clear binding signals in the BLI assays ( **new Fig. S10B** and **10E**, the third panels).

These new results confirm that several RBD-Fc variants that did not show detectable ACE2 binding in BLI assays, but exhibited cell-cell fusion activity, are able to bind the corresponding ACE2 when weak interactions are amplified by higher binding valency. Valency dependent avidity amplification also explains higher sensitivity to weak interactions in cell-based assays.

New binding results using S-trimer variants have been added to the updated Supplementary Fig. 10 (as shown below):

We have added a description of these results in the text in MS Lines 374-404.

“Consistently, the BL change conferred hACE2 mediated pseudovirus entry, while pseudovirus entry mediated by the cognate bACE2*_R.cor_* reduced by ~52-fold (**Fig. 4M**). Despite conferring detectable cell-cell fusion activity and pseudovirus entry, hACE2 binding by the dimeric BL variant of the Rc-o319 RBD-Fc protein remained below the detection limit of the BLI assay, while bACE2*_R.cor_* binding was markedly reduced and fell below the detection limit of the BLI assay (**Fig. 4B, first panel**). A subsequent BLI assay using the BL variant of the Rc-o319 S-trimer detected weak hACE2 binding (**Fig. S10B**), likely rendered detectable by amplification through trivalent binding-mediated avidity, underscoring a dependence of assay sensitivity on binding valency. As cell-based assays involve highly multivalent interactions mediated by multiple S-trimers and receptors, avidity-enhanced binding renders them substantially more sensitive to weak interactions (also see the Limitations section).”

We have also added a *Limitations* section to briefly describe the above discussion of possible reasons underlying discrepancies between biophysical BLI assays and cell-based assays:

Lines 800-843 in MS:

“A limitation of this study is that the biophysical BLI assays employed relatively low-valency analytes (monomeric RBD, dimeric ACE2, or trimeric S-protein), thereby limiting avidity-mediated binding amplification and sensitivity to weak interactions. In contrast, cell-cell fusion and pseudovirus entry assays (Figs. 4, 5, and S16) involve highly multivalent interactions between multiple surface-displayed S-trimers and ACE2 molecules, resulting in substantially enhanced avidity and sensitivity. Accordingly, several interactions that were undetectable by BLI nonetheless supported detectable fusion or entry in cell-based assays (Figs. 4B, 4F, 4G, 4I, 4K, 4L, and 5). A further limitation is that cellular phenotypes can be influenced by S-protein-related factors beyond receptor binding, potentially including S-protein stability, efficiency of surface expression, incorporation into pseudovirus particles, and activation by cellular proteases. This is exemplified in Fig. 5, where the SARS-CoV-2 S-protein exhibited the strongest fusion activity mediated by both WT bACE2*_R.cor_* and the glycan mutant bACE2*_R.cor_*-ΔGLC_38_, despite no detectable binding to either receptor in BLI assays. An additional example is shown in Fig. S16, where a SARS-CoV-1 S-protein variant carrying the Rc-o319 RBM (SARS1_Rc-o319RBM_) failed to mediate bACE2*_R.cor_*-dependent fusion despite detectable expression, suggesting incompatibility between the swapped RBM and the S-protein backbone. While cell-based assay readouts often scale with large differences in receptor-binding affinity, they may not scale linearly when affinity differences are modest and the above-discussed S-protein–related factors are involved. Several RBM-modified S-proteins exhibited modestly increased ACE2 binding in BLI assays but reduced fusion or entry activity in cell-based assays (Fig. 4A vs D and Fig. 4G vs H). We speculate that RBM changes may affect S-protein stability, surface presentation, or interactions with host factors, leading to nonlinear relationships between receptor-binding affinity and cellular phenotype. Finally, future studies using animal models will be required to assess the relevance of receptor-affinity changes in the context of infection.”

3. In terms of binding to hACE2, is the BL loop or the RBM loop of the RBD more critical for receptor recognition? The conclusions drawn in the Results and Discussion sections regarding the relative importance of these structural elements could be more clearly articulated to better reflect the experimental findings.

**Response:**

We thank the reviewer for this important comment. The relative importance between RBM-loop and BL loop change in Rc-o319 S-protein binding to hACE2 can be first considered structurally. As shown in Fig. 3J, the RBM-loop of SARS-CoV-2 does not contact directly with hACE2. Instead, it stabilizes the conformation of the large-loop (LL)/beta-loop (BL) and indirectly contributes to the LL/BL region in the ACE2 binding interface.

Further, we showed that replacing the BL in Rc-o319 with the SARS-CoV-2 large loop (LL) confers hACE2-mediated cell-cell fusion. This result is further consistent with a previous report, which demonstrated that the SARS-CoV-2 large loop is essential for hACE2 engagement and that its deletion eliminates detectable hACE2 binding and pseudovirus entry (Fig.3 in PMID: 39402048). Together with this previous report, our structural data and the BL to LL loop swapping result gave the conclusion that changing the BL loop to SARS-CoV-2 LL (the BL change) is the essential requirement for hACE2 recognition, whereas the RBM loop likely only fine-tunes the interaction mediated by the BL/LL region.

We performed extra mutagenesis experiments and carried out cell-cell fusion assays to further clarify the roles of the BL loop and the RBM-loop in hACE2 recognition by Rc-o319. Consistently, in the new data, introducing the RBM-loop change (W430F_Rc-o319_+S432K_Rc-o319_) alone was not sufficient to confer hACE2 usage in cell-cell fusion assays (new **Supplementary Fig. 8B**). In contrast, the BL change alone was sufficient to confer hACE2-mediated cell-cell fusion (**Fig. 4B**), demonstrating that the BL loop represents the minimal structural requirement for productive hACE2 engagement in a cellular context. Collectively, our data show that the RBM-loop change functions to further enhance or stabilize hACE2 binding when combined with the BL change, but does not independently determine receptor usage in a cellular context.

New Supplementary Figure 8:

In the revised manuscript, we have clarified the specific roles of the BL and RBM-loop in conferring hACE2 usage in both the *Results and Discussion* sections (lines 469-481 and 704-716).

Lines 469-481 in the track-changes MS:

“To further clarify the role of the RBM-loop in hACE2 recognition, we introduced the RBM-loop change alone into the Rc-o319 S-protein. This change did not confer hACE2-dependent cell-cell fusion (**Fig. S8B**), consistent with structural analyses indicating that the RBM-loop does not make direct contacts with ACE2 (**Fig. 3**). No detectable hACE2 binding was observed for the Rc-o319 BL-variant RBD-Fc (**Fig. 4B**), and a similar lack of hACE2 binding was observed for the Rc-o319 BL+LM-variant RBD-Fc in BLI assays (**Fig. 4I**), despite the corresponding S-proteins mediating clear hACE2-dependent cell-cell fusion and pseudovirus entry (**Fig. 4B, I** and **M**). When the RBM-loop change was introduced into the Rc-o319 BL- or BL+LM-variant RBD-Fc (i.e., BL+RBM-loop and BL+LM+RBM-loop variants), weak but detectable hACE2 binding could be detected in BLI assays (**Fig. 4B, I-K**). These results confirm that the RBM-loop plays a modulatory role, essential for mediating high-affintiy ACE2 interaction.”

Lines 704-716 of the track-changes MS:

“Consistently, swapping the BL with the LL of SARS-CoV-2 (the BL change) alone enabled detectable hACE2-mediated cell-cell fusion activity, even though hACE2 binding by BL variants remained undetectable using RBD-Fc (**Fig. 4B**) or only very weak using S-trimer (**Fig. S10B**) in BLI assays. Combined BL and RBM-loop substitutions enabled Rc-o319 RBD-Fc binding to both hACE2 and bACE2*_R.cor_* to be detected by BLI, albeit with very weak affinities (**Fig. 4B** and **J**). These results highlight the importance of the BL/LL region together with its corresponding RBM-loop structure in ACE2 recognition, as well as the adaptation of the Rc-o319 BL and RBM-loop structures to bACE2*_R.cor_*. Cell-cell fusion assays showed that the BL change alone was sufficient to confer hACE2-mediated fusion, whereas the RBM-loop change alone was insufficient (**Fig. S8B** and **C**), indicating that changes within the RBM BL region constitute the primary determinants enabling hACE2 utilization (**Fig. 4** and **Fig. S8**).”

4. Why does the bACE2R.cor-ΔGLC38 mutation enhance the binding affinity of Rc-o319 while simultaneously reducing its cell fusion efficiency? How do these findings shed light on the functional role of the Asn38-glycan at the interaction interface?

**Response:**

We thank the reviewer for this comment. We apologize for the confusion caused. The confusion likely stems from suboptimal fluorescence intensity in the original images, which may have given the wrong impression that the bACE2*_R.cor_*-ΔGLC_38_ receptor reduced cell-cell fusion efficiency of Rc-o319. We have replaced the image in the **revised** **Fig. 5C Rc-o319 panel** to show that bACE2*_R.cor_*-ΔGLC_38_ mediated comparable or enhanced fusion by the Rc-o319 S-protein.

In fact, our cell-cell fusion assay data actually showed that the removal of the Asn38-glycan generally enhanced fusion activity mediated by diverse sarbecovirus S-proteins. The bACE2*_R.cor-_*ΔGLC_38_ variant receptor not only slightly enhanced fusion by Rc-o319 but also slightly enhanced fusion by Clade 1b SARS-CoV-2, BANAL-20-52, BANAL-20-236 and RaTG13 S-proteins. Of note, the bACE2*_R.cor_-*ΔGLC_38_ variant receptor mediated much more pronounced enhancement of fusion activity for SARS-CoV-1 (Clade 1a) and BtKY72 (Clade 3) S-proteins (**revised** **Fig. 5B-C, derived from old Fig. 4I-J**).

BLI assay results show that the removal of the Asn38-glycan was able to enhance bACE2*_R.cor_* binding not only for Rc-o319 but also for the BANAL-20-52 and BANAL-20-236 S-trimers (**Fig. S11A, C** and **E**). In particular, although unable to utilize WT bACE2*_R.cor_*, BtKY72 S-protein, with a type-2 RBM, of a clade 3 sarbevirus, was able to utilize bACE2*_R.cor_*-ΔGLC_38_, albeit exhibiting only very weak cell-cell fusion activity. Consistently, BLI binding results show that the BtKY72 S-trimer was unable to bind bACE2*_R.cor_*, but gained bACE2*_R.cor_*-ΔGLC_38_ binding with an estimated affinity of 11.8 nM (**Fig. S11H, the second panel**).

Regarding the functional role of the Asn38-glycan, although structural analysis shows that the Asn38-linked glycan of bACE2*_R.cor_* forms specific interactions with Rc-o319 RBD residues F423_Rc-o319_, Y426_Rc-o319_, H444_Rc-o319_, and N459_Rc-o319_, our data indicate that this glycan generally dampens bACE2*_R.cor_* utilization by sarbecovirus S-proteins, including for Rc-o319, likely through steric effects that reduce S-protein binding. Introduction of an Asn38-linked glycan into bACE2*_Ra_*_9479_ also dampened its usage by SARS-CoV-1 and BtKY72 S-proteins (**Fig. S16**). We noted that, in cell-cell fusion assays, the Asn38-linked glycan had a greater impact on S-proteins mediating weak to intermediate fusion activity than on those with strong fusion activity, suggesting that it may exert a more pronounced effect on viruses with limited entry capacities.

For improved clarity, we added further description and discussion of the Asn38-linked glycan in both the *Results* and *Discussion* sections in lines 525-555 and 751-786, respectively.

Lines 525-555 of the track-changes MS of *Results* section:

“In cell-cell fusion assays, the bACE2*_R.cor_*-ΔGLC_38_ variant receptor showed a tendency to mediate enhanced fusion activities for all the sarbecovirus S-proteins capable of utilizing the WT bACE2*_R.cor_* **(Fig. 5B-C)**. BLI assay results show that the removal of the Asn38-glycan was able to enhance bACE2*_R.cor_* binding not only for Rc-o319 but also for the BANAL-20-52 and BANAL-20-236 S-trimers (**Fig. S11A**). In particular, although unable to utilize WT bACE2*_R.cor_*, BtKY72 S-protein, with a Type-2 RBM, of a clade 3 sarbevirus, was able to utilize bACE2*_R.cor_*-ΔGLC_38_, albeit exhibiting only very weak cell fusion activity. Consistently, BLI binding results show that the BtKY72 S-trimer was unable to bind bACE2*_R.cor_*, but gained bACE2*_R.cor_*-ΔGLC_38_ binding with an estimated affinity of 11.8 nM (**Fig. S11H, the second panel)**.”

Lines 751-786 of the track-changes MS *Discussion* section:

“Further, although structural analysis shows that the Asn38-linked glycan of bACE2*_R.cor_* forms specific interactions with Rc-o319 RBD residues F423_Rc-o319_, Y426_Rc-o319_, H444_Rc-o319_, and N459_Rc-o319_, our data indicate that this glycan generally dampens bACE2*_R.cor_* utilization by sarbecovirus S-proteins, including for Rc-o319, likely through steric effects that reduce S-protein binding. Introduction of an Asn38-linked glycan into bACE2*_Ra_*_9479_ also dampened its usage by SARS-CoV-1 and BtKY72 S-proteins. We noted that, in cell-cell fusion assays, the Asn38-linked glycan had a greater impact on S-proteins mediating weak to intermediate fusion activity than on those with strong fusion activity (**Fig. 5**). We speculate that this glycan may exert a more pronounced restrictive effect on viruses with limited entry capacities. Interestingly, the Asn38-linked glycan is also positioned near the binding interface of HKU5-like, ACE2-utilizing merbecovirus S-proteins and may therefore modulate their receptor binding (58, 59). ”

To facilitate understanding of Asn38-glycan on different clades of sarbecoviruses with different types of RBDs, RBM types are now shown alongside virus clades in the Updated Fig. 5 (shown below).

Reviewer #2:

Major comments

1. Figure 4: The results suggest that the swapping of BL of Rc-o319 allows to use human ACE2 for cell-cell fusion, but not for ACE2 binding. On the other hand, Rc-o319 BL+SL+LM+AL+RBM-loop variant and Rc-o319-RBD-RBMSARS2 variant showed both cell fusion activity and ACE2 binding affinity. To clarify the effect of substitutions in RBM-loop and BL for ACE2 binding of Rc-o319, cell-cell fusion assay and BLI using Rc-o319 RBD BL+RBM-loop variant are recommended.

**Response:**

Thank you for your valuable comments and suggestions. As recommended, we have now included additional cell-cell fusion assay, pseudovirus entry assay and BLI binding assay results for the Rc-o319 BL+RBM-loop variant in the revised Figure 4J and 4M (shown below) to further clarify specific contributions of the BL and RBM-loop structures to ACE2 binding in Rc-o319 S-protein.

New updated Figure 4:

For the newly included set of experiments involving the BL+RBM-loop variant, hACE2 binding by BLI was detected, albeit with very weak affinity (**Fig. 4J**). This new result shows that the BL+RBM-loop change represents the minimal set of changes sufficient to confer detectable hACE2 binding in BLI assays using dimeric RBD-Fc.

To further clarify the roles of the BL loop and the RBM-loop in hACE2 recognition in Rc-o319, extra mutagenesis experiments show that introducing the RBM-loop change (W430F_Rc-o319_+S432K_Rc-o319_) alone was not sufficient to confer hACE2 usage in cell-cell fusion assays (new **Supplementary Fig. 8**). However, the BL change alone was sufficient to enable hACE2-mediated cell-cell fusion (**Fig. 4B**). Together, these results demonstrate that the BL provides the primary structural framework required for ACE2 compatibility, whereas the RBM-loop fine-tunes receptor binding efficiency and specificity.

New Supplementary Figure 8:

We have added the following comments about the BL+RBM-loop and related variants, in both the *Results* and the *Discussion* sections.

lines 469-481 of the track-changes MS:

“To further clarify the role of RBM-loop in hACE2 recognition, the RBM-loop change alone was introduced in the Rc-o319 S-protein, showing an inability to utilize hACE2 for cell-cell fusion (**Fig. S8B**). No detectable hACE2 binding was observed for the Rc-o319 BL-variant RBD-Fc (**Fig. 4B**), and a similar lack of hACE2 binding was observed for the Rc-o319 BL+LM-variant RBD-Fc in BLI assays (**Fig. 4I**), despite the corresponding S-proteins mediating clear hACE2-dependent cell-cell fusion and pseudovirus entry (**Fig. 4B, I** and **M**). When the RBM-loop change was introduced into the Rc-o319 BL- or BL+LM-variant RBD-Fc (i.e., BL+RBM-loop and BL+LM+RBM-loop variants), weak but detectable hACE2 binding could be detected in BLI assays (**Fig. 4B, I-K**). These results confirm that the RBM-loop plays a modulatory role, essential for mediating high-affinity ACE2 interaction.”

Lines 704-716 of the track-changes MS *Discussion* section:

“Consistently, swapping the BL with the LL of SARS-CoV-2 (the BL change) alone enabled detectable hACE2-mediated cell-cell fusion activity, even though hACE2 binding by BL variants remained undetectable using RBD-Fc (**Fig. 4B**) or only very weak using S-trimer (**Fig. S10B**) in BLI assays. Combined BL and RBM-loop substitutions enabled Rc-o319 RBD-Fc binding to both hACE2 and bACE2*_R.cor_* to be detected by BLI, albeit with very weak affinities (**Fig. 4B** and **J**). These results highlight the importance of the BL/LL region together with its corresponding RBM-loop structure in ACE2 recognition, as well as the adaptation of the Rc-o319 BL/RBM-loop structures to bACE2*_R.cor_*. Cell-cell fusion assays showed that the BL change alone was sufficient to confer hACE2-mediated fusion, whereas the RBM-loop change alone was insufficient (**Fig. S8B** and **C**), indicating that the BL change is the dominant determinant for hACE2 utilization (**Fig. 4** and **Fig. S8**).”

2. Figure 4: There is the explanation of the reason that Rc-o319 RBD BL variant showed cell fusion activity in human ACE2 but not binding affinity for human ACE2. However, the reason why Rc-o319 RBD AL variant showed high binding affinity for bACE2R.cor but cell fusion activity using the ACE2 was decreased is not explained in this manuscript. This explanation is recommended to be added to Discussion section.

**Response:**

We thank the reviewer for this important comment. To ensure the robustness of our results, we repeated the experiments in the original Figure 4 and additionally performed pseudovirus entry assays, as you suggested. The updated figure shows results that are consistent with our original findings. We briefly discuss possible reasons for several discrepancies between receptor binding assays and cell-based assays.

The reason that BL variant showed activities mediated by hACE2 in cell-based assays but no detectable binding to hACE2 in biophysical BLI assays was primarily due to different sensitivities of the two types of assays relating to binding valency. In biophysical BLI assays, relative low-valency analytes (monomeric RBD, dimeric ACE2, or trimeric S-trimer) were used, thereby restricting avidity-mediated binding amplification and limiting sensitivity to weak interactions. In contrast, cell-cell fusion and pseudovirus entry assays (Figs. 4, 5, and S16) involve highly multivalent interactions between multiple surface-displayed S-trimers and ACE2 molecules, resulting in substantially enhanced avidity and sensitivity.

In the revised manuscript, we used a trimeric BL-variant S-trimer to assay hACE2 binding and detected a binding signal, albeit weak. The increased avidity-amplified binding resulting from trivalent binding, compared with the previous divalent binding, enabled detection of binding by BLI. (Please also refer to the answer to point 2 of reviewer #1)

Regarding the discrepancy between the BLI assay results and the cell-based assay results for the AL variant, we repeated the BLI assays, cell-cell fusion assays and performed additional pseudovirus entry assays. These results showed the same overall trend as observed previously, namely that although the AL variant increased receptor binding in BLI assays, this increase did not translate proportionally into enhanced fusion or entry in cell-based assays.

We speculate that RBM changes may affect S-protein stability, surface presentation, or interactions with host factors, such as proteases, leading to nonlinear relationships between receptor-binding affinity and cellular phenotype.

We have added the above explanation to the *Results* section (lines 359-367) of the track-changes MS.

Lines 359-367 in track-changes MS:

“Interestingly, the AL change very slightly increased bACE2*_R.cor_* binding when assayed using both Rc-o319-RBD-Fc and the Rc-o319 S-trimer (**Fig. 4D, the first panels** and **Fig. S9E** and **S14F**). The discrepancy between the increased BLI affinity and the reduced cellular activity may reflect differences in assay conditions. In the cellular context, phenotypes may also be influenced by S-protein-related factors beyond receptor binding, such as S-protein stability, efficiency of surface expression, incorporation into pseudovirus particles, and activation by cellular proteases, which could contribute to the observed discrepancy (see the Limitations section).”

Additionally, we have also added a Limitations section to briefly describe the above considerations that may underlie these discrepancies between biophysical BLI assays and cellular assays.

“A limitation of this study is that the biophysical BLI assays employed relatively low-valency analytes (monomeric RBD, dimeric ACE2, or trimeric S-protein), thereby limiting avidity-mediated binding amplification and sensitivity to weak interactions. In contrast, cell-cell fusion and pseudovirus entry assays (Figs. 4, 5, and S16) involve highly multivalent interactions between multiple surface-displayed S-trimers and ACE2 molecules, resulting in substantially enhanced avidity and sensitivity. Accordingly, several interactions that were undetectable by BLI nonetheless supported detectable fusion or entry in cell-based assays (Figs. 4B, 4F, 4G, 4I, 4K, 4L, and 5). A further limitation is that, in the cellular context, phenotypes can be influenced by S-protein-related factors beyond receptor binding, potentially including S-protein stability, efficiency of surface expression, incorporation into pseudovirus particles, and activation by cellular proteases. This is exemplified in Fig. 5, where the SARS-CoV-2 S-protein exhibited the strongest fusion activity mediated by both WT bACE2*_R.cor_* and the glycan mutant bACE2*_R.cor_*-ΔGLC38, despite no detectable binding to either receptor in BLI assays. An additional example is shown in Fig. S16, where a SARS-CoV-1 S-protein variant carrying the Rc-o319 RBM (SARS1_Rc-o319RBM_) failed to mediate bACE2*_R.cor_*-dependent fusion despite detectable expression, suggesting incompatibility between the swapped RBM and the S-protein backbone. While cell-based assay readouts often scale with large differences in receptor-binding affinity, they may not scale linearly when affinity differences are modest and the above-discussed S-protein-related factors are involved. Several RBM-modified S-proteins exhibited modestly increased ACE2 binding in BLI assays but reduced fusion or entry activity in cell-based assays (Fig. 4A vs D and Fig. 4G vs H). We speculate that RBM changes may affect S-protein stability, surface presentation, or interactions with host factors, leading to nonlinear relationships between receptor-binding affinity and cellular phenotype. Finally, future studies using animal models will be required to assess the relevance of receptor-affinity changes in the context of infection.”

3. In this study, not only computational modelling or cryo-EM, but also binding affinity validation using GFP-fusion assay and BLI. However, it may strengthen the manuscript to include more binding affinity validation like pseudovirus entry assay. Otherwise, the authors should discuss limitations the lack of pseudovirus entry assay.

**Response:**

We thank the reviewer for raising this important point. We agree that pseudovirus entry assays provide valuable and complementary functional validation of receptor usage beyond structural modeling, cryo-EM analysis, and in vitro BLI binding assays. In response to this suggestion, we have now included VSV-based pseudovirus entry assays for the Rc-o319 S-protein and its variants in 293T cells over-expressing TMPRSS2.

Specifically, we performed pseudovirus entry assays using both bACE2*_R.cor_* and hACE2 as receptors, and these data have been incorporated into the revised manuscript (new **Fig. 4M**). Notably, the pseudovirus entry results are largely consistent with the corresponding GFP-based cell-cell fusion assays.

For the AL mutant, pseudovirus entry was reduced, consistent with the cell-cell fusion results. We speculate that, in the cellular context, phenotypes may be influenced by S-protein-related factors beyond receptor binding, potentially including S-protein stability, efficiency of surface expression, incorporation into pseudovirus particles, and activation by cellular proteases. We now discuss these factors in our updated *Discussion* and *Limitation* sections.

**(M)** Pseudovirus assay. Entry of VSV particles pseudotyped with Rc-o319 S-protein variants into HEK293T cells transiently expressing hACE2 or bACE2*_R.cor_* and co-expressing TMPRSS2. Luciferase activity was measured 24 h post-transduction. Data are presented as mean ± s.d., n = 3. The *P* values were determined by unpaired t-test. **P* < 0.05, ***P* < 0.01, ****P* < 0.001 and *****P* < 0.0001.

VSV-pseudovirus entry assay results were added in track-changes MS lines 367-397:

“Changing the LM region alone (the LM change) reduced bACE2*_R.cor_* binding and utilization (**Fig. 4C, the first, third** and **fifth panels)** but still failed to confer hACE2-mediated fusion activity and pseudovirus entry (**Fig. 4C, the fourth** and **fifth panels**).

Swapping of the Rc-o319 BL region to the LL sequence of SARS-CoV-2-RBD (the BL change), appears to confer Rc-o319 with the ability to utilize hACE2 for cell fusion activity at the expense of substantially reduced fusion activity mediated by its cognate bACE2*_R.cor_* receptor (**Fig. 4B**). Consistently, the BL change conferred hACE2 mediated pseudovirus entry, while pseudovirus entry mediated by the cognate bACE2*_R.cor_* reduced by ~52-fold (**Fig. 4M**). Despite conferring detectable cell-cell fusion activity and pseudovirus entry, hACE2 binding by the dimeric BL variant of the Rc-o319 RBD-Fc protein remained below the detection limit of the BLI assay, while bACE2*_R.cor_* binding was markedly reduced and fell below the detection limit of the BLI assay (**Fig. 4B, the first panels**).”

Lines 415-418 in track-changes MS:

“However, unexpectedly, the BL+LM+AL+SL variant, designed to introduce optimal interactions across all four RBM regions, exhibited enhanced hACE utilization in cell-cell fusion and pseudovirus-entry assays, yet its hACE2 binding remained too weak to be detected by BLI.”

Accordingly, the VSV-Pseudovirus entry assay method was added in the *Method* section (track-changes MS lines 1036-1053).

Reviewer #3:

1. Do the types of S-proteins described in the paper group co-pylogenetically? It’s a bit confusing to read this nomenclature alongside the also widely used phylogenetic Clade 1-5 descriptions.

**Response:**

We appreciate this helpful comment. Our classification is structurally defined and is consistent with RBM deletion features: type-1 RBDs are the most complete and lack deletions of more than one amino acid; type-2 RBDs contain deletions of 2-5 AA in the small loop; and type-3 RBDs contain large deletions in both the large and small loops. As a result, each RBD type exhibits distinct structural features. We find that describing sarbecovirus S-proteins using our previously defined type-1 to type-3 framework is useful for understanding receptor binding, as it abstracts away broader genetic factors underlying clade or lineage classifications and focuses specifically on RBD regions responsible for receptor engagement. For example, within this framework, S-proteins from both clade 1a and clade 1b sarbecoviruses are unified as possessing type-1 RBDs, consistent with their typically broader ACE2 binding range.

With respect to the phylogenetic relationship between the two classification schemes, when referenced against the clade system previously defined by Hu et al. (PMID: 29190287), type-1 RBDs correspond to clade 1a/b viruses, type-3 RBDs to clade 2 viruses, and type-2 RBDs to clade 3 viruses, indicating that the two classification systems are largely co-phylogenetic. Prompted by the reviewer’s comment, we have revised our classification of the Rc-o319 RBD, designating it as type-1b rather than the previously proposed type-4. This places Rc-o319 alongside other type-1 S-proteins within clade 1, while the type-1b designation highlights its distinctive β-loop structure arising from a 9-amino-acid deletion in the region corresponding to the canonical large loop of type-1 RBDs, rendering it structurally distinct and reflecting a unique adaptation to bACE2*_R.cor_*.

To avoid confusion, we have clarified this framework in the revised manuscript and updated Figure 5 to explicitly map the type-1 to type-3 RBD classification onto sarbecovirus clades 1a, 1b, 2, and 3.

Updated Fig. 5:

In addition, to further clarify the relationships among the spike proteins analyzed in this study, we have added a sarbecovirus phylogenetic tree based on the amino acid sequences of 72 representative sarbecovirus S-proteins using the MERS-CoV S-protein as the outgroup in the new Supplementary Fig. 2.

In the main text, we have also described our previously defined type-1-3 RBD classification in detail in the Introduction. The relationship between the structurally defined RBD types and the genetically defined clade classification is now clearly elaborated in lines 75-89 and 93-106.

Lines 75-89 in the track-changes MS :

“Phylogenetic classification of SARSr-CoV based on S-RBD sequences has defined multiple clades (34). Clade 1 sarbecoviruses utilize ACE2 and can be further subdivided into clade 1a (SARS-CoV-1 lineage) and clade 1b (SARS-CoV-2 lineage), both of which possess the most complete RBMs, lacking deletions of more than one amino acid. In contrast, no cellular receptor has been identified for clade 2 strains, which harbor deletions at two distinct regions within the RBM. Finally, clade 3 viruses contain a single loop deletion within the RBM and exhibit variable capacities for ACE2 utilization. Based on these distinct RBM deletion patterns and their associated structural features among clades 1-3 viruses, we previously classified their RBDs as type-1, type-3, and type-2, respectively (17).”

Lines 93-106 in the track-changes MS:

“Type-1 RBD S-proteins, including those from clade 1a and 1b sarbecoviruses such as SARS-CoV-1, SARS-CoV-2 and WIV1, possess intact LL and SL regions. Type-1 RBD S-proteins often bind hACE2, albeit with varying affinities: SARS-CoV-1 and SARS-CoV-2 bind hACE2 with high affinities, whereas WIV1 binds hACE2 with a moderate affinity (35). We found high-affinity hACE2 binding requires synergistic favourable hACE2 interactions across the four featured RBM regions. Type-2 RBD S-proteins, including those from clade 3 sarbecoviruses such as BM48-31 and BtKY72, have an intact LL but a deleted SL. They generally lack hACE binding but can bind selected bat ACE2 (bACE2) orthologs with intermediate affinities. Type-3 RBD S-proteins, including those from clade 2 sarbecoviruses such as HeB2013 and RmYN02, have both their LL and SL regions deleted. To date, type-3 RBD S-proteins have not been reported to bind either hACE2 or bACE2.”

2. Is it known where in the cell the linoleic acid, oleic acid and biliverdin molecules are acquired to permit Spike locking?

**Response:**

We thank the reviewer for raising this important question. The precise cellular stage at which these small molecules are acquired is not yet known. Based on the occluded nature of the linoleic acid-binding sites within the locked S-trimer, we speculate that linoleic acid binding likely occurs in the endoplasmic reticulum (ER) during S-protein synthesis or early maturation. This is consistent with the ER being a major site of fatty acid metabolism. In contrast, biliverdin binds within the NTD at a more exposed site, suggesting that it could bind to the S-protein either during biosynthesis or after secretion from the cell.

Notably, although linoleic acid binding has been proposed to stabilize locked conformations by mediating extra monomer-monomer interactions within the S-trimer, we and others have previously determined structures of sarbecovirus S-proteins that lack linoleic acid-like molecules bound yet still adopt locked conformations (PMID: 40085715; PMID: 37507385; PMID: 33707453; PMID: 32647346). Similarly, locked S-trimer structures have also been observed in the absence of biliverdin binding. These in vitro observations suggest that linoleic acid binding is not strictly required for adoption of the locked conformation. Instead, we have proposed that an acidic milieu, such as that in the ER, may play an important role in promoting or stabilizing the locked state (PMID: 35905112).

3. Figure 1A: Given the results of these experiments it would be useful to understand the sequence heterogeneity in the ACE2 that is responsible for the lack of wide bat ACE2 usage and whether that is the same restriction imposed on human ACE2 binding. This could also be brought in later (see point 6 below).

**Response:**

We agree with the reviewer that sequence heterogeneity in ACE2 is a major determinant dictating its recognition by sepcific sarbecovirus S-proteins, and that analogous molecular determinants likewise govern hACE2 recognition.

Using our RBM interaction-region framework (BL/LL, LM, AL, and SL), mutagenesis analyses indicate that the affinity of the Rc-o319 S-protein for bACE2*_R.cor_* is largely dictated by the BL and LM regions, as combined changes in these regions rendered BLI binding undetectable. In contrast, changes in the AL and SL regions had little impact on bACE2*_R.cor_* binding.

For hACE2 recognition, we found that the combined BL+LM+RBM-loop changes conferred only weak binding affinity. Intermediate to high affinity was achieved only when BL, LM, AL, SL, and RBM-loop changes were introduced together (BL+LM+AL+SL+RBM-loop), suggesting that both the AL+SL change is required to support hACE2 affinity.

With respect to the restricted usage of bACE2 orthologs by the Rc-o319 S-protein, although bACE2*_Ra_*_9479_, bACE2*_Ra_*_5539_, and bACE2*_Rs_*_1446_ supported very weak fusion activity, none exhibited detectable binding to Rc-o319 RBD-Fc in BLI assays. We speculate that the specialized BL structure contributes to this specificity, based on structural analysis and sequence comparison. In the Rc-o319 RBD:bACE2*_R.cor_* complex, Lys27 of bACE2*_R.cor_* contacts RBM-loop residue W430_Rc-o319_ and BL residue Q448_Rc-o319_, while Glu75 of bACE2*_R.cor_* forms a salt bridge with BL residue K454_Rc-o319_ (Fig. 3A and F). Both Lys27 and Glu75 are highly specific in bACE2*_R.cor_* among bACE2 orthologs (new Supplementary Figure 17), and their specialized interactions with the Rc-o319 BL structure likely contribute to the preferential usage of bACE2*_R.cor_* by Rc-o319 S-protein.

New Supplementary Figure 17:

The following comments are added to the *Discussion* Section about the specialized interaction between bACE2*_R.cor_* and Rc-o319 S-protein BL region.

Lines 664-691 in the track-changes MS:

“Notably, Lys27 is uniquely present in bACE2*_R.cor_* and bACE2*_R.ferr_* among related ACE2 molecules. However, whereas most ACE2 molecules contain a Glu75 residue, bACE2*_R.ferr_* instead contains a Lys75, which would abolish the K454_Rc-o319_-Glu75 salt bridge observed in our Rc-o319-RBD:bACE2*_R.cor_* complex structure (**Fig. 3A** and **F**) and likely introduce electrostatic repulsion with the BL residue K454_Rc-o319_. The specific interaction pattern between Rc-o319-RBD-BL and bACE2*_R.cor_* demonstrates an adaptation of the Rc-o319 RBM region to its cognate bACE2*_R.cor_* receptor.”

Lines 788-791 in the track-changes MS:

“ In summary, the complementary interactions between the specialized RBM, particularly the BL region, of the Rc-o319 S-protein and the corresponding interacting residues in bACE2*_R.cor_* appears to reflect a highly specific virus-host co-adaptation (**Fig. S17** and **Table S10**).”

4. Lines 184-186: “Because the NTD is under strong immune pressure (47, 48), these truncated and elongated loops likely endow the NTD of Rc-o319 S-protein with a distinct antigenic profile.” These references are for SARS2 where our understanding of immune selection pressure is significant. I don’t doubt there is also selection pressure on the NTD in bats but it seems a bit early to definitively state this to be the case.

**Response:**

We fully agree with the reviewer that the cited references primarily reflect immune selection pressure acting on the NTD of SARS-CoV-2, where antigenic pressure has been extensively characterized. We also agree that, although immune pressure on the NTD likely exists in bat sarbecoviruses, little experimental evidence can support a definitive conclusion.

Accordingly, in the revised manuscript, we have clarified that the discussion of strong immune pressure specifically refers to the NTD of SARS-CoV-2, thereby avoiding overstatement. We now state that the truncated and elongated loops in the NTD of Rc-o319 may contribute to a distinct antigenic profile, without explicitly attributing this feature to strong immune selection pressure in bats.

Lines 243-247 of the track-change MS:

“It’s well-established that the SARS-CoV-2 S-protein NTD is under strong immune pressure (49, 50). The truncated and elongated loops observed in the Rc-o319 S-protein NTD may therefore confer a distinct antigenic profile, although whether these features reflect immune-driven selection in bats remains to be determined.”

5. Figure 4A-F etc. It's not clear for the described mutants if these proteins are stable. Can you include the confirmatory gels to clear this up. I can see the Western in Figure S11 but the expression seems very variable – can you just confirm equal amounts were loaded onto probes for BLI etc.

**Response:**

We thank the reviewer for raising this important point regarding protein stability and loading consistency. We acknowledge that the expression levels of full-length S-proteins in 293T cells can vary, and we have now included a Limitations section discussing how this variability may affect cell-based assays. However, this variability does not affect the biophysical BLI measurements presented in Fig. 4.

All BLI assays shown in Fig. 4 were performed using purified RBD-Fc proteins rather than full-length S-proteins. For each BLI experiment, equal amounts of RBD-Fc (11 μg/mL) were immobilized on Protein A sensors, ensuring comparable ligand loading and consistent signal normalization across all measurements. This experimental design minimizes potential effects arising from variable protein expression levels that may influence cell-based assays. To further address concerns regarding protein quality and stability, we have included SDS-PAGE analyses of the purified RBD-Fc proteins used for the BLI experiments in the new Supplementary Fig. 15. These analyses confirm comparable purity and integrity among all RBD-Fc variants tested.

**Figure S15. SDS-PAGE of non-reducing and reducing samples of different Rc-o319 RBD-Fc proteins (10 ug) used for BLI experiments.**

6. Figure 4: Novelty and conclusions. It’s not so surprising that a total chimera of the RBM would finally switch the functional phenotype between the cornutus and human ACE2. And the slightly contradictory results for the R.cor ACE2 glycan mutant (that its removal amplifies fusion) means it remains unclear why Rc-o319 has evolved the distinct RBM structures described. Could the authors try more mutants of other Spikes (e.g. introducing Rc-o319 changes to the SARS1 clade viruses, and seperately, comparing and mutating related Rhinophulus ACE2s (9479 and non users) to explain this in more detail). It seems to me that from a novelty perspective it would be good to ultimately understand what is it that drives the selection process.

**Response:**

We thank the reviewer for this thoughtful comment regarding novelty and evolutionary interpretation. We agree that a complete RBM swap, on its own, is not unexpected to alter ACE2 usage. However, in this study we went beyond RBM swapping to identify specific regions and residues that are critical for switching receptor usage.

Specifically, we found that while the BL change alone was sufficient to confer measurable hACE2-mediated cell-based fusion and entry activity to the Rc-o319 S-protein, detectable hACE2 binding by BLI was only achieved through the combined BL and RBM-loop changes. Notably, in addition to the BL change representing a replacement of the specialized BL structure in the Rc-o319 RBM with the LL in the SARS-CoV-2 RBM, we identified two specific residues within the RBM loop that stabilize the LL conformation required for high-affinity hACE2 binding. Conversely, retaining the specialized BL structure is critical for efficient bACE2*_R.cor_* binding. The molecular determinants governing ACE2 specificity and affinity identified here represent novel findings of this study.

Together with a systematic analysis of additional point mutations in other RBM regions (LM and SL), our study dissects the contributions of individual regions and residues to ACE2 binding in Rc-o319 S-protein RBM. In this context, the RBM-swapping experiment was used primarily as a positive control and reference, rather than as the central source of novelty.

Our survey of sarbecovirus S-proteins for their ability to bind and utilize bACE2*_R.cor_* (Figs. 5 and S11) further showed that, although bACE2*_R.cor_* can mediate fusion activity for several sarbecovirus S-proteins, most of these interactions were too weak to be detected by BLI assays, even when S-trimers enabling avidity-enhanced trivalent binding were tested. Among the S-trimers examined, only BANAL-20-236 exhibited detectable, albeit weak, binding to bACE2*_R.cor_*. By comparison, binding of the SARS-CoV-2 S-trimer to bACE2*_R.cor_* was undetectable by BLI, despite its RBM being closely related to that of BANAL-20-236. Notably, these two RBMs differ at the LM region, where SARS-CoV-2 contains a neutral residue (Q493_SARS2_). Consistent with this observation, introduction of the corresponding positive-to-neutral LM change in Rc-o319 (K458Q_Rc-o319_) markedly reduced both binding and fusion activity (Fig. 4C), while introducing a positively charged LM region in SARS-CoV-2 RBD-Fc conferred detectable bACE2*_R.cor_* binding (Fig. S9L).

More broadly, most type-1 RBD S-proteins from clade 1a and 1b sarbecoviruses possess a neutral LM region (PMID: 40085715), and our mutagenesis results suggest that a neutral LM is unfavorable for affinity toward bACE2*_R.cor_*. Although type-2 RBD S-proteins from clade 3 viruses often harbor positively charged LM regions, they nevertheless exhibit even weaker utilization of bACE2*_R.cor_* probably due to their RBM deletions. In contrast, we show that the specialized BL structure of the Rc-o319 S-protein is a key determinant underlying its relatively high-affinity binding to bACE2*_R.cor_*. Likely, without this distinctive BL structure, most sarbecovirus S-proteins display very limited bACE2*_R.cor_* usage in cell-cell fusion assays. These findings therefore support a specific adaptation of the Rc-o319 S-protein to bACE2*_R.cor_*.

In addition, our glycan mutagenesis experiments in both bACE2*_R.cor_* and bACE2*_Ra_*_9479_ (new Fig. S16, as suggested by the reviewer) demonstrate that the Asn38-linked glycan generally hinders ACE2 utilization by S-proteins. Notably, in cell-cell fusion assays, this glycan exerted a greater inhibitory effect on S-proteins mediating weak to intermediate fusion activity than on those with strong fusion activity, suggesting a more pronounced restrictive role for viruses with limited binding and entry capacity.

Taken together, these observations suggest that the evolved, specialized BL loop of the Rc-o319 S-protein, together with additional structural features, confers preferential affinity for bACE2*_R.cor_*, consistent with a specialized adaptation of Rc-o319. Further, the presence of the Asn38-linked glycan and other sequence features of bACE2*_R.cor_* appear to restrict its utilization by most other sarbecovirus S-proteins that lack strong affinity for this receptor.

New Supplementary Figure 16 testing the effect of Asn38-glycan on bACE2*_Ra_*_9479_:

Lines 556-574 in the track-changes MS:

“To further assess the functional role of the Asn38 glycan, an N-linked glycosylation site was introduced into a *Rhinolophus affinis* bat ACE2(bACE2*_Ra_*_9479_), by substituting Asp38 with Asn, generating bACE2*_Ra_*_9479_-GLC_38_. In both cell-cell fusion and BLI assays, BtKY72 and SARS-CoV-1 S-proteins exhibited substantially reduced fusion activity utilizing bACE2*_Ra_*_9479_-GLC_38_ compared with the WT bACE2*_Ra_*_9479_ receptor (**Fig. S16B** and **F; S11J** and **K**). In parallel, we swapped the RBMs of SARS-CoV-1 and BtKY72 S-proteins with that of Rc-o319, generating chimeric SARS1_Rc-o319RBM_ and BtKY72_Rc-o319RBM_ S-proteins. Although being expressed by the cell (**Figure S16I** and **J**), the RBM swapping appeared to render the SARS1_Rc-o319RBM_ S-protein non-functional, unable to mediate cell-cell fusion mediated by either bACE2*_Ra_*_9479_ or bACE2*_R.cor_* receptor and their Asn38-glycan variants (**Fig. S16C** and **D**). The BtKY72_Rc-o319RBM_ S-protein showed substantially reduced utilization of the bACE2*_Ra_*_9479_ and bACE2*_Ra_*_9479_-GLC_38_ receptors (**Fig. S16F** and **H**). In contrast, its utilization of both bACE2*_R.cor_* and bACE2*_R.cor_*-ΔGLC_38_ was only marginally enhanced compared with the WT BtKY72 S protein, yet remained substantially weaker than that of the Rc-o319 S protein **(Fig. S16E** and **G** and **Fig. 4A)**. These results highlight compatibility constraints between different RBMs and S-protein backbones, indicating that RBM changes can adversely affect overall S-protein function. ”

Lines 751-784 in the track-changes MS:

“Further, although structural analysis shows that the Asn38-linked glycan of bACE2*_R.cor_* forms specific interactions with Rc-o319 RBD residues F423_Rc-o319_, Y426_Rc-o319_, H444_Rc-o319_, and N459_Rc-o319_, our data indicate that this glycan generally dampens bACE2*_R.cor_* utilization by sarbecovirus S-proteins and reduces binding, including for Rc-o319, likely through steric effects. Introduction of an Asn38-linked glycan into bACE2*_Ra_*_9479_ also dampened its usage by SARS-CoV-1 and BtKY72 S-proteins. We noted that, in cell-cell fusion assays, the Asn38-linked glycan had a greater impact on S-proteins mediating weak to intermediate fusion activity than on those with strong fusion activity **(Fig. 5)**. We speculate that this glycan may exert a more pronounced restrictive effect on viruses with limited entry capacities.”

7. Discussion lines 440: “Therefore, bACE2R.cor appears to have evolved to permit efficient binding by the S-proteins of highly specific sarbecovirus species.” I’m not sure I understand this sentence – is that positive or negative selection by the virus on its host?

**Response:**

Our data support the interpretation that bACE2*_R.cor_* is selectively permissive to only a narrow subset of sarbecoviruses with highly specialized RBMs, rather than broadly facilitating usage by diverse sarbecoviruses. Consistent with this, bACE2*_R.cor_* exhibits negative selectivity toward most sarbecovirus clades, suggesting an evolutionary trajectory in which this receptor broadly restricts sarbecovirus infection (Fig. 5). Rc-o319 represents a notable exception, having evolved a distinct receptor-binding strategy involving specialized RBM features (including the BL and RBM-loop regions) that enable relatively high-affinity binding to bACE2*_R.cor_* and efficient fusion. Together, these observations are most consistent with virus-driven adaptation to a restrictive host receptor, rather than positive selection imposed by the host.

In summary, the interaction between the Rc-o319 S-protein, defined by a specialized RBM and its distinctive BL region, and the corresponding BL-interacting residues in bACE2*_R.cor_* exemplifies a highly specific co-adaptation between the virus and its host receptor.

For clarity, we changed the description in lines 748-751 in the track-changes MS:

“Although multiple S-proteins were capable of mediating bACE2*_R.cor_*-dependent cell-cell fusion, most exhibited little or no detectable bACE2*_R.cor_* binding in BLI assays, suggesting that the Rc-o319 S-protein has evolved a uniquely specialized specificity and affinity for bACE2*_R.cor_*.”

**Part III – Minor Issues: Editorial and Data Presentation Modifications** Please use this section for editorial suggestions as well as relatively minor modifications of existing data that would enhance clarity.

Reviewer #1: 1.It would be beneficial to provide an earlier introduction to the phylogenetic relationship between Rc-o319 and other Sarbecoviruses, as well as the diversity of RBD types, to better contextualize the significance of the findings.

**Response:** We thank the reviewer for this suggestion and we have made the following changes in our main text:

Lines 75-89 of the track-changes MS:

“Phylogenetic classification of SARSr-CoV based on their S-RBD sequences has led to the definition of multiple clades (34). Clade 1 sarbecoviruses comprise ACE2-using viruses and can be further divided into clades 1a (SARS-CoV-1 lineage) and 1b (SARS-CoV-2 lineage), both of which possess the most complete RBMs, lacking deletions of more than one amino acid. In contrast, no cellular receptor has been identified for clade 2 sarbecoviruses, which harbor deletions at two distinct regions within the RBM. Finally, clade 3 viruses contain a single loop deletion within the RBM and exhibit variable capacities for ACE2 utilization. Based on these distinct RBM deletion patterns and their associated structural features among clades 1-3 viruses, we previously classified their RBDs as type-1, type-3, and type-2, respectively (17).”

Lines 111-115 of the track-changes MS:

“The nucleotide and amino acid sequences of Rc-o319 were more homologous to those of viruses belonging to the SARS-CoV-2 lineage (Clade 1b) than the SARS-CoV-1 lineage (Clade 1a) within the *Sarbcecovirus* subgenus (**Fig. S2**).”

Reviewer #2: Minor comments 1. Material and Method: The authors showed Western Blot data (Figure S11A and S11C). Therefore, the explanation of the method of Western Blot should be added to the section.  2. The authors should provide additional details about computational tools (e.g. versions of CryoSPARC, Chimera)

**Response:** We thank the reviewer for this suggestion and we have made the following changes in our track-changes MS:

We have added the Western Blot and Flow cytometry methods to the *Methods* section (lines 1055–1093).

In addition, information on the CryoSPARC and Chimera versions has been added to the *Methods* section.

Lines 976-977 in the track-changes MS:

“For the Rc-o319-RBD:bACE2*_R.cor_* complex dataset, data processing was performed mainly using cryoSPARC v4.6.2 (67).”

Lines 999-1003 in the track-changes MS:

“Model building and adjustment were performed manually in Coot 0.9 (68). Steric clash and sidechain rotamer conformations were improved using the Namdinator web server (69). Final structures after additional manual adjustments were refined and validated in PHENIX-1.18.261 (70).”

Lines 1005-1007 in the track-changes MS:

“The starting model was fitted in the cryo-EM density in Chimera 1.18.0 before manual adjustments were carried out in Coot 0.9.”

Reviewer #3: n/a see main summary for all other points.
