# Supplementary material for: Structural basis for sarbecovirus Rc-o319 spike adaptation to Rhinolophus cornutus Bat ACE2 and constraints on switching to human ACE2
Source: PLoS Pathog. 2026 May 21;22(5):e1014245. doi: 10.1371/journal.ppat.1014245 (PMC13232947; doi:10.1371/journal.ppat.1014245)
Supplement: Attachment — Submitted filename: Response to Reviewer_PPATHOGENS-D-25.docx [file ppat.1014245.s031.docx]

**Additional Editor Comments :**

**With respect to Reviewer 3, comment 1: please see the type I, II, III, and IV nomenclature defined by Si et al. Nat Comms 2024 (PMID 39402048). It would be better for the field as a whole to use this established nomenclature rather than introduce yet another redundant system with different relationships between “type x” and the indel structure. Si et al.'s nomenclature also has the advantage of using roman numerals which better distinguishes from the arabic numeral evolutionary clade designations.**

**Response:**

We thank the reviewer and editor for this suggestion. To ensure consistency with the field, we have now adopted the type I–IV nomenclature defined by Si et al. (Nat Commun, 2024) throughout the revised manuscript.

Our previous classification (types 1–3) of sarbecovirus RBDs was reported in ***Science Advances*** (DOI: 10.1126/sciadv.adr8772), in which we showed that sarbecovirus RBDs differ substantially in their extended loop domains, leading to the classification into three types. Specifically, type-1 RBDs retain full-length extended loop domains, type-2 RBDs harbor a deletion in the small loop (SL), and type-3 RBDs lack both the “SL” and the disulfide bond-stabilized large loop (LL).

To maintain continuity with our previous work, we provide a clear correspondence between the two classification systems at their first occurrence (type I = type-1, type II = type-2, etc.), and subsequently use the Si et al. nomenclature consistently throughout the manuscript.

In this framework, Rc-o319 RBD, which contains a nine-amino acid deletion in the large loop (LL) that is critical for ACE2 engagement in SARS-CoV-1 and SARS-CoV-2, is classified as a type III RBD according to Si et al.

**We have revised the *Introduction* section accordingly to clarify this classification (Lines 104-111), as follows:**

“Similarly, the type I–IV (Roman numerals) classification of sarbecovirus RBDs proposed by Si et al. is based primarily on indel patterns within the RBM(28). In this scheme, type I RBDs correspond to our type-1 and retain a full-length RBM; type II RBDs correspond to our type-2 and contain a single deletion in the small loop; type III RBDs harbor a deletion in the large loop; and type IV RBDs correspond to our type-3 and feature two deletions in RBM. Accordingly, Rc-o319 RBD, with a 9-amino-acid deletion in large loop, was defined by Si et al as a type III RBD (28).”

**Please address the additional editor comment from the initial decision letter:**

**“In the form-fields outside of the written reviews below, one reviewer noted the DURC potential of the finding of mutations that endow human ACE2 tropism to this bat sarbecovirus (though the mutations are of course found and tested in safe experimental systems). The authors could consider discussing the cost-benefit analysis that favors publication of this result in the Discussion and/or Methods.”**

**Response:** We appreciate the reviewer’s comment regarding the potential DURC(Dual Use Research of Concern) of this work.

First, the experimental systems used in this study, including pseudovirus entry and cell-cell fusion assays, are well-established, widely adopted in the field, and do not involve replication-competent viruses, ensuring a high level of biosafety. In addition, the use of coordinated substitutions to modulate hACE2 binding has been previously described (Science Advances, PMID: 40085715).

Second, although certain substitutions can enhance hACE2 binding in cell-based systems, these effects may not directly translate to authentic viral infection, where additional constraints, such as viral fitness, spike stability and entry efficiency, play critical roles.

In addition, the Rc-o319 mutant analyzed here was generated by introducing combinations of substitutions (BL, LM, AL, SL and RBM-loop) derived from SARS-CoV-2, rather than representing novel or artificially designed mutations. The resulting RBD shares 85% sequence identity with that of SARS-CoV-2, indicating that these substitutions fall within a naturally occurring sequence space rather than creating new sequence features. Accordingly, these changes are unlikely to introduce fundamentally new antigenic determinants, but instead recapitulate sequence characteristics already present in circulating sarbecoviruses.

The key benefit of this work is the identification of coordinated, multi-regional constraints governing receptor adaptation, demonstrating that efficient adaptation cannot be achieved through individual mutations alone but instead requires concerted changes across the Rc-o319 RBD. This reveals substantial structural and evolutionary barriers to hACE2 utilization. Notably, regions beyond the canonical hACE2-binding interface, including the RBM loop, actively contribute to receptor engagement, highlighting the integrated nature of these interactions and further reinforcing these constraints.

These insights provide a mechanistic framework for identifying structural and evolutionary barriers that limit zoonotic emergence, which is essential for risk surveillance and preparedness. Therefore, the benefits of improving our understanding of receptor adaptation and cross-species transmission substantially outweigh potential risks.

Taken together, this work does not facilitate Rc-o319 viral adaptation; rather, it defines the mechanistic and structural barriers to hACE2 usage, enabling a more accurate assessment of zoonotic risk. All experiments were conducted in accordance with established biosafety guidelines.

**We have revised the *Limitations of this study* section accordingly to clarify this classification (Lines 677-683), as follows:**

“The effects observed in cell-based systems may not fully recapitulate authentic viral infection, where additional biological constraints and host factors come into play. Addressing this will require studies with authentic viruses and in vivo models to evaluate how receptor-affinity changes influence infection in a physiological context. Despite these limitations, our findings delineate key barriers to receptor adaptation and provide a conceptual framework for assessing cross-species transmission risk.”
